# Recruitment of Pontin/Reptin by E2f1 amplifies E2f transcriptional response during cancer progression

**DOI:** 10.1038/ncomms10028

**Published:** 2015-12-07

**Authors:** Amy Tarangelo, Nathanael Lo, Rebecca Teng, Eunsun Kim, Linh Le, Deborah Watson, Emma E. Furth, Pichai Raman, Ursula Ehmer, Patrick Viatour

**Affiliations:** 1 Division of Cancer Pathobiology, Department of Pathology and Laboratory Medicine, Center for Childhood Cancer Research, Children's Hospital of Philadelphia, Philadelphia, Pennsylvania 19104, USA; 2 Department of Biomedical and Health Informatics, Children's Hospital of Philadelphia, Philadelphia, Pennsylvania 19104, USA; 3 Department of Pathology and Laboratory Medicine, Perelman School of Medicine, University of Pennsylvania, Philadelphia, Pennsylvania 19104, USA; 4 School of Biomedical Engineering, Sciences and Health Systems, Drexel University, Philadelphia, Pennsylvania 19104, USA; 5 Department of Medicine, University Hospital of Munich, Munich 81675, Germany

## Abstract

Changes in gene expression during tumorigenesis are often considered the consequence of *de novo* mutations occurring in the tumour. An alternative possibility is that the transcriptional response of oncogenic transcription factors evolves during tumorigenesis. Here we show that aberrant E2f activity, following inactivation of the Rb gene family in a mouse model of liver cancer, initially activates a robust gene expression programme associated with the cell cycle. Slowly accumulating E2f1 progressively recruits a Pontin/Reptin complex to open the chromatin conformation at E2f target genes and amplifies the E2f transcriptional response. This mechanism enhances the E2f-mediated transactivation of cell cycle genes and initiates the activation of low binding affinity E2f target genes that regulate non-cell-cycle functions, such as the Warburg effect. These data indicate that both the physiological and the oncogenic activities of E2f result in distinct transcriptional responses, which could be exploited to target E2f oncogenic activity for therapy.

## Abstract (web summary)

E2F transcription factors are primarily known for the regulation of the cell cycle and are often dysregulated in cancer. Here, the authors show that during cancer progression E2F1 recruits a Pontin/Reptin complex to E2F target genes to open chromatin and increase E2F transcriptional response.

## Graphical abstract

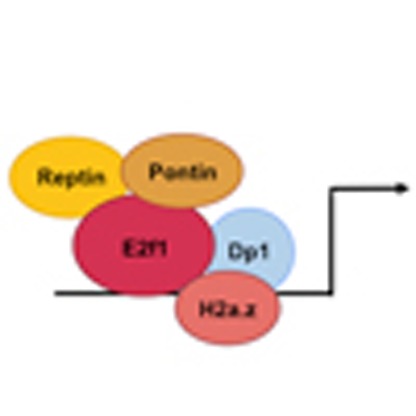

The E2f family of transcription factors is composed of activator (E2f1-3a) and repressor (E2f3b, 4–8) factors and is predominantly regulated by the Rb family of proteins (Rb, p107 and p130)^1^,^2^,^3^,^4^. Activator E2f1-3 display high conservation of their DNA-binding domains, and previous studies indicate that they mostly share overlapping target genes. In contrast, other domains of E2f1-3 are poorly conserved among the family, suggesting that activator E2fs could interact with distinct co-factors and thereby modulate their activity in a context-dependent manner. However, the identity of these co-factors as well as the consequences of their recruitment in a variety of physio-pathological contexts is poorly understood^5^.

Under physiological conditions, Rb proteins maintain cellular quiescence by binding and repressing the transcriptional activity of E2f1-3. Mitogenic stimuli-induced stabilization of Cyclin/CDK complexes and the subsequent phosphorylation of Rb proteins disrupt the Rb/E2f physical interaction, thereby derepressing E2f factors and promoting the transactivation of genes associated with cell cycle and an exit from quiescence^6^,^7^,^8^. The role of E2f factors in the regulation of cell cycle is particularly well established, but evidence indicates that E2f factors also regulate non-cell-cycle functions under specific conditions. However, how and in what contexts E2f factors regulate these mostly unknown non-cell-cycle functions remains unclear^1^.

Genetic or epigenetic events leading to inactivation of the Rb family and unrestricted transcriptional activity of E2f factors are almost universal in cancer^9^. Besides sustained proliferation, the consequence of aberrant activity of E2f1-3 on cancer initiation and progression remains poorly defined, limiting the development of effective therapies to specifically target oncogenic E2f activity. In addition to alterations targeting upstream components of the Rb pathway, activator E2fs are also overexpressed in tumours. Of interest to this study, E2f1 is frequently found overexpressed in several types of cancer, including lung cancer, melanoma and hepatocellular carcinoma (HCC)^1^. Data from previous studies suggest that increased E2f1 expression promotes the progression of these cancers, but the mechanism is mostly unknown^10^.

HCC is a devastating disease characterized by common alterations in the Rb pathway following HBV/HCV (hepatitis B and C viruses) chronic infection and other genetic events, as well as overexpression of E2f1 (ref. 11). We have previously determined that ablation of the Rb gene family in the liver of adult mice (triple knock out-cTKO mice) triggers the development of HCC (TKO HCC) that recapitulates many histological and molecular characteristics of the human disease^11^. In this study, we have taken advantage of this model to determine the role of E2f factors and the mechanisms that modulate their transcriptional response during cancer progression. Our results show that E2f1 recruits the Pontin/Reptin complex to open chromatin structure at E2f target genes and amplify their transactivation by E2f factors during TKO HCC progression. They introduce the concept that E2f transcriptional response evolves during cancer progression, including the activation of target genes that control non-cell-cycle functions such as the regulation of glucose metabolism, also known as the Warburg effect.

## Results

### E2f factors regulate the Warburg effect in liver cancer

**Figure 1:**
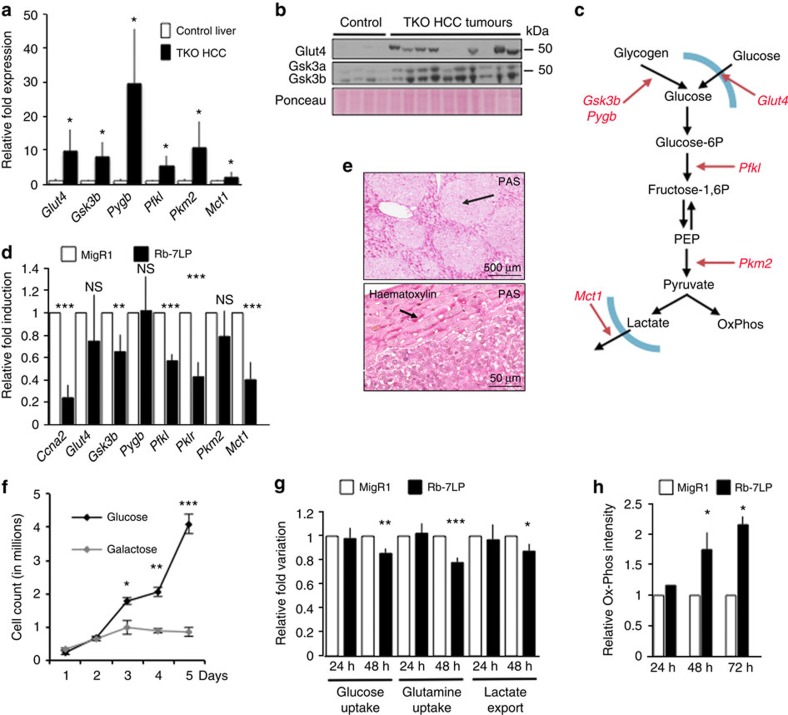
Rewiring of glucose metabolism in TKO HCC. (**a**) Reverse transcription–quantitative PCR (RT–qPCR) analysis in control liver (*n*=5) and TKO HCC (*n*=9) for *Glut4*, *Gsk3b*, *Pygb*, *Pfkl*, *Pkm2* and *Mct1*. (**b**) Glut4 and Gsk3a/b expression, as detected by immunoblotting in control livers (*n*=4) and TKO HCC (*n*=10). Loading was normalized by Ponceau staining. (**c**) Activation of a set of genes (in red) that collectively regulate multiple aspects of glucose metabolism in TKO HCC. PEP, Phosphoenolpyruvate. (**d**) RT–qPCR analysis of *Ccna2* (*Cyclina2*) and metabolic target genes expression in TKO HCC cells 24 h after transduction with a MigR1-IRES-GFP retrovirus either empty (MigR1, white bars) or expressing Rb-7LP (black bars; *n*=3). (**e**) Periodic acid–Schiff (PAS) staining of TKO HCC shows absence of glycogen in tumour zones, whereas adjacent non-tumour zones display normal presence of glycogen. Upper panel: low magnification of PAS staining (black arrow indicates a tumor zone). Lower panel: high magnification of PAS staining, counterstained with haematoxylin staining (black arrow indicates glycogen). (**f**) Growth curve of TKO HCC cells cultured with media containing either 4.5 g l^−1^ of glucose or galactose (*n*=3). (**g**,**h**) Metabolites concentration (**g**) and ROS production (**h**) in TKO HCC cells 24, 48 or 72 h after infection with empty (white) or Rb-7LP (black)-expressing MigR1-IRES-GFP retrovirus (*n*=3), error bars represent standard deviation. *P<0.05; **P<0.01; ***P<0.001. NS, not significant.

To identify novel functions driven by E2f factors during TKO HCC development, we performed a computational analysis of the recently published TKO HCC transcriptome^11^ and identified a set of genes (*Glut4*, *Pygb*, *Gsk3b*, *Pkm2*, *Pfkl* and *Mct1*) that displays increased expression in TKO HCC. We validated this finding by reverse transcription–quantitative PCR for all six genes and immunoblotting for Glut4 (an average of 12.64-fold induction compared with controls) and Gsk3a/b (an average of 8.35- and 2.69-fold induction compared with controls for a and b, respectively) to confirm that these genes are transactivated in TKO HCC (Fig. 1a,b). These genes regulate multiple aspects of glucose metabolism (Fig. 1c) and will be described hereafter as metabolic target genes. To establish the role of E2f factors in the regulation of metabolic target genes in TKO HCC, we derived two cell lines from independent TKO HCC primary tumours (hereafter TKO HCC cells) to serve as an experimental system. Repression of E2f transcriptional activity by introduction of a stabilized form of Rb (Rb-7LP)^12^ (Supplementary Fig. 1a) led to decreased expression of metabolic target genes (Fig. 1d), indicating that E2f factors regulate their expression in TKO HCC. In contrast, genes regulating the tricarboxylic acid cycle were largely unaltered in TKO HCC (Supplementary Fig. 1b).

To test the consequence of increased expression of metabolic target genes on glucose metabolism in TKO HCC, we detected glycogen by periodic acid–Schiff base staining. We found that TKO HCC tumour zones are depleted of glycogen in contrast to neighbouring non-tumour zones, thereby correlating with increased *Gsk3b* and *Pygb* expression (Fig. 1e). Substitution of glucose with galactose (which bypasses glycolysis and promotes oxidative phosphorylation^13^) in the culture media of TKO HCC cells impaired their proliferation, indicating that glycolysis is critical for TKO HCC proliferation (Fig. 1f). Finally, Rb-7LP expression in TKO HCC cells led to decreased glucose and glutamine uptake^14^ as well as lactate export (Fig. 1g); in parallel, glucose metabolism shifted towards oxidative metabolism without an increase of the mitochondrial biomass (Fig. 1h; Supplementary Fig. 1c). These data show that E2F factors activate a set of genes that alter glucose metabolism by promoting glycolysis and lactate export (a phenomenon known as the Warburg effect) in TKO HCC.

### Distinct waves of E2f target genes during cancer progression

**Figure 2:**
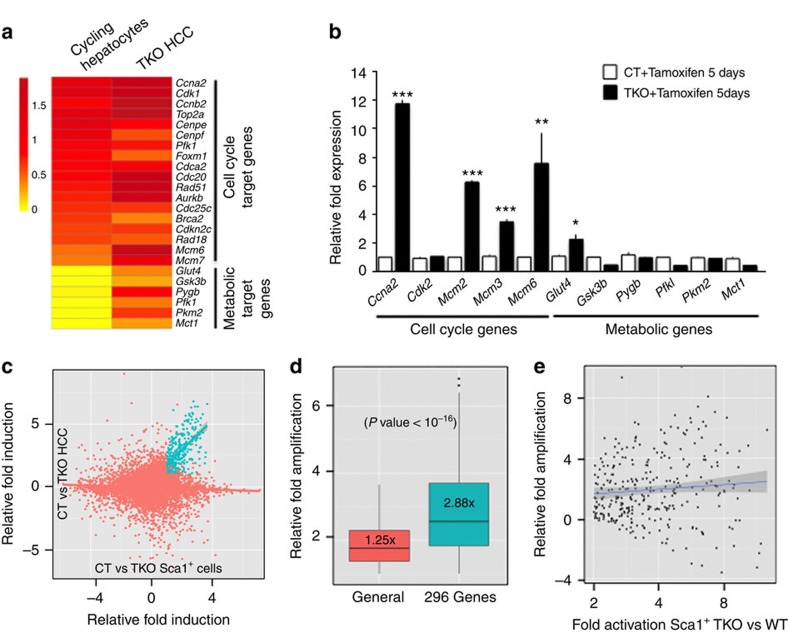
Amplification of E2f transcriptional response during TKO HCC progression. (**a**) Relative fold expression of metabolic target genes and a representative set of cell cycle genes upon transient entry of hepatocytes into cell cycle (cycling hepatocytes) and in TKO HCC, compared with their respective controls. Fold induction is displayed as log2-based intensity ratios. (**b**) Reverse transcription–quantitative PCR analysis of five representative cell cycle genes (*Ccna2*, *Cdk2*, *Mcm2*, *Mcm3* and *Mcm6*) and metabolic target genes in control and *Rosa26Cre-ER cTKO* liver, 5 days after Tamoxifen treatment-induced Rb family loss (*n*=3). (**c**) Comparison of array analysis performed in early and late lesions: fold induction in TKO versus control Sca1^+^ cells is displayed on the *x* axis, whereas fold induction in TKO HCC versus control liver is displayed on the *y* axis. Blue dots represent the 296 genes transactivated in both data sets (at least two-fold induction and *P*-value<0.05; *P*-value obtained by *t*-test). Other genes are represented by red dots. Blue and Red lines represent average expression for their respective populations. Fold induction is displayed as log2-based intensity ratios. (**d**) Average amplification of transactivation levels from early to late lesions. Left: average amplification for the general group (all genes transactivated at least twofold in either early or late lesions). Right: 296 common target genes group, as identified by blue dots in **c** (E2f target genes). The box represents the data included within the 25th to 75th percentile. The horizontal bar represents the average. *P*-value obtained by *t*-test. (**e**) Amplification of the transcriptional activation for the 296 genes (black dots) from early to late lesions (*y* axis) is plotted against transactivation level in TKO Sca1^+^ cells versus CT Sca1^+^ cells (*x* axis). The black line represents the average amplification, whereas the grey zone represents the error margin. The horizontality of the line indicates that the transcriptional amplification is, on average, similar for all genes without any correlation for their transactivation level in early lesions. Error bars represent standard deviation *P<0.05; **P<0.01; ***P<0.001.

This result is unexpected given that alteration of glucose metabolism upon transient exit from quiescence, which is characterized by robust E2f transcriptional activity, has not been previously reported. To determine whether metabolic target genes are activated in the context of transient cell cycle activity, we analysed their expression in primary hepatocytes, either quiescent or transiently progressing through the cell cycle (as identified by the expression of the CyclinB1-green fluorescent protein (GFP) transgene^15^), isolated from the liver of 3.5-week-old mice. Although proliferative hepatocytes display a robust cell cycle programme activation compared with their quiescent counterparts, they failed to display any transactivation of metabolic target genes expression (Fig. 2a and Supplementary Data 1). To test the activation of metabolic target genes upon short-term inactivation of Rb family genes, we isolated primary hepatocytes from control and *Rosa26-CreER*^*T2*^ *TKO* mice after five consecutive daily injection of Tamoxifen. Cell cycle genes rapidly displayed significant transactivation (albeit at a lower level of transactivation than was observed in TKO HCC (Supplementary Fig. 2a)). In contrast, metabolic target genes were not transactivated in these conditions, with the exception of Glut4, which displayed limited transactivation (Fig. 2b). These data suggest a progressive mode of E2f activity: an initial (‘early') wave of cell cycle genes is rapidly activated by E2f factors. Following prolonged E2f activity, another (‘late') wave of target genes, composed of genes with non-cell-cycle functions, such as the regulation of glucose metabolism, is progressively activated by E2f factors.

To test this hypothesis and determine the evolution of E2f transcriptional response during TKO HCC progression, we took advantage of our previous identification of two distinct stages of TKO HCC development: (i) early lesions composed of non-transformed Sca1^+^ progenitor cells that progressively evolve into (ii) late lesions composed of well-differentiated tumour cells^11^. We compared array analysis performed at each stage (Supplementary Fig. 2b and Supplementary Data 2) and found striking differences in gene expression profiles. Among the 17,306 genes expressed at both stages of TKO HCC progression, only a limited set of 296 genes (highly enriched for E2f target genes with cell cycle functions (*P*-value<10^−86^; *P*-value obtained by *T*-test); Supplementary Data 3) is commonly upregulated (Fig. 2c, blue dots), suggesting that proliferation is the only shared feature between both stages (Supplementary Fig. 2c,d). We found that the average general transactivation (defined for each stage as the average fold transactivation for all genes activated at least 2-fold) is only amplified 1.25-fold from early to late lesions (an average fold transactivation of 5.35 in early lesions versus 6.67 in late lesions). In contrast, the average transactivation of the 296 genes is amplified 2.88-fold from early to late lesions (an average fold transactivation of 4.1 in early lesions versus 11.8 in late lesions; Fig. 2d, *t*-test). Importantly, this amplification occurs in a linear manner (Fig. 2e), indicating that its underlying mechanism is independent of the intensity of the initial transactivation occurring in early lesions.

Based on these computational analyses, we hypothesized that a set of E2f-regulated target genes is specifically amplified during TKO HCC progression. To test this hypothesis, we first investigated the possibility that amplification of E2f transcriptional response is the consequence of decreased activity of repressor E2f4-8. We found that E2f4-6 expression is unchanged while E2f7-8 expression is increased in TKO HCC, as expected since they are themselves E2f target genes^16^ (Supplementary Fig. 2e,f). In addition, we found no variation in the binding intensity of E2f4 to metabolic target genes in TKO HCC (Supplementary Fig. 2g), suggesting that the amplification of E2f transcriptional response in TKO HCC does not primarily originate from decreased activity of repressor E2fs.

### E2f1 recruits Pontin and Reptin

**Figure 3:**
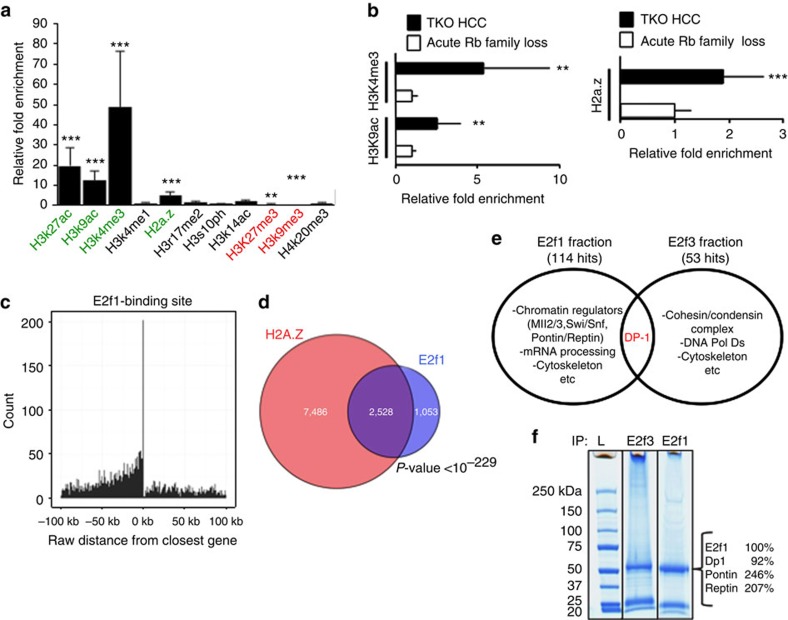
Accumulation of the H2a.z histone variant at E2f target genes in TKO HCC. (**a**) ChIP assay to detect the enrichment of common epigenetic marks at the promoter region of the panel of E2f target genes (see Fig. 2b for gene list) in TKO HCC cells, as determined by quantitative PCR (qPCR) analysis (*n*=3). Green: marks positively enriched; red: marks negatively enriched. (**b**) ChIP assay to detect the enrichment for the H3k9ac and the H3k4me3 marks (left) as well as the H2a.z histone variant (right) at the promoter region of the panel of E2f target genes in TKO HCC (black bar, long term Rb family loss) and in Rosa26 Cre-ER^T2^ TKO liver 4 days after Tamoxifen treatment (white bar, acute Rb family loss), as determined by qPCR analysis (*n*=3). The relative fold enrichment is compared for both conditions with the fold enrichment observed in control liver and arbitrarily set at 1 for the Acute Rb family loss condition. (**c**) ChIP-Seq was performed to identify the genomic sequences bound by E2f1 in TKO HCC. The graph determines the distance of E2f1 bound regions from the closest gene. (**d**) Venn diagram showing the overlap between E2f1 and H2a.z-bound genes in TKO HCC. *P*-value obtained by hypergeometric test. (**e**,**f**) E2f1 and E2f3 were pulled-down from TKO HCC lysates and the pull-down fractions were allowed to migrate on an acrylamide gel subsequently stained with Coomassie Blue for visualization (represented in **f**) and processed by mass spectrometry (*n*=3). The relative abundance of E2f1 (arbitrarily set at 100%), Dp1 (positive control) and Pontin/Reptin in the E2f1 pull-down fraction is indicated on the right of the display. All these proteins were isolated from the same band migrating at ∼50 kDa. (**e**) Summary of the principal findings of the MS performed with E2f1 and E2f3 in TKO HCC. The complete results are displayed in Supplementary Data 5. Error bars represent standard deviation *P <0.05; **P<0.01; ***P<0.001.

Based on these observations, we next hypothesized that activator E2fs increase E2f transcriptional response by recruiting a novel epigenetic mechanism during TKO HCC progression. To test this hypothesis, we utilized two parallel and complementary approaches. First, we screened for the enrichment of a series of histone modifications in TKO HCC cells and in primary TKO HCC at a panel of E2f target genes (composed of the 11 genes detected in Fig. 2b). We found that H3k27ac, H3k9ac, H3k4me3 (which are commonly associated with active transcription)^17^,^18^ are enriched in the regulatory regions of E2f target genes in TKO HCC cells. This result is not surprising given the increased transactivation of E2f target genes described above. More surprisingly, we also found that H2a.z, a histone variant for H2a associated with destabilization of chromatin and increased transcription^19^,^20^, is enriched in the regulatory regions of E2f target genes (Fig. 3a,b and Supplementary Fig. 3a). To determine the frequency of H2a.z enrichment at E2f target genes in TKO HCC, we performed chromatin-immunoprecipitation sequencing (ChIP-Seq) analysis with H2a.z and E2f1 pull-down fractions from TKO HCC cells, which resulted in the identification of 10,014 and 3,581 unique hits genome-wide, respectively. As expected, the majority of hits in the E2f1 ChIP-Seq were found within or in close proximity to intergenic regions (Fig. 3c, Supplementary Fig. 3b and Supplementary Data 4). In addition, H2a.z and E2f1 hits very significantly overlapped (Fig. 3d), suggesting that enrichment for the H2a.z histone variant is a common feature of E2f target genes in TKO HCC.

In a second parallel approach to identify epigenetic regulators interacting with activator E2fs in TKO HCC, we performed mass spectrometry (MS) in the pull-down fraction of E2f1 and E2f3 in TKO HCC cells (Fig. 3e,f). Using this unbiased strategy, we found that E2f1 co-precipitated with 114 proteins, including multiple components of the Mll2/3 and Swi/Snf complexes, whereas E2f3 co-precipitated with 53 proteins. Strikingly, we found that the only common protein in the interactomes of E2f1 and E2f3 is Dp1, suggesting distinct functions for E2f1 and E2f3 in TKO HCC (Fig. 3e and Supplementary Data 5). Importantly, we found that E2f1, but not E2f3, specifically interacts with Pontin and Reptin (also designated as Ruvbl1 and Ruvbl2; Fig. 3f). Pontin and Reptin are putative DNA helicases with homology to the bacterial Ruvb protein. They are involved in multiple cellular processes such as transcription, DNA repair and chromatin decondensation. Importantly, Pontin and Reptin form a homo/heterocomplex of 6 or 12 subunits that is both necessary and sufficient for H2a/H2a.z exchange in nucleosomes^21^,^22^. Finally, the Pontin/Reptin complex can be either found alone or included in larger complexes such as Tip60 and Srcap^23^,^24^. However, our MS approach as well as ChIP assay for Tip60 protein in TKO HCC cells failed to identify a role for these larger complexes in TKO HCC, suggesting that E2f1 recruits isolated Pontin/Reptin complexes in TKO HCC (Supplementary Fig. 3c and Supplementary Data 5). Therefore, the combination of our mini ChIP-screen and MS approaches suggests that E2f1 recruits a Pontin/Reptin complex to integrate H2a.z and destabilize chromatin at E2f target genes, resulting in the amplification of E2f transcriptional response in TKO HCC.

**Figure 4:**
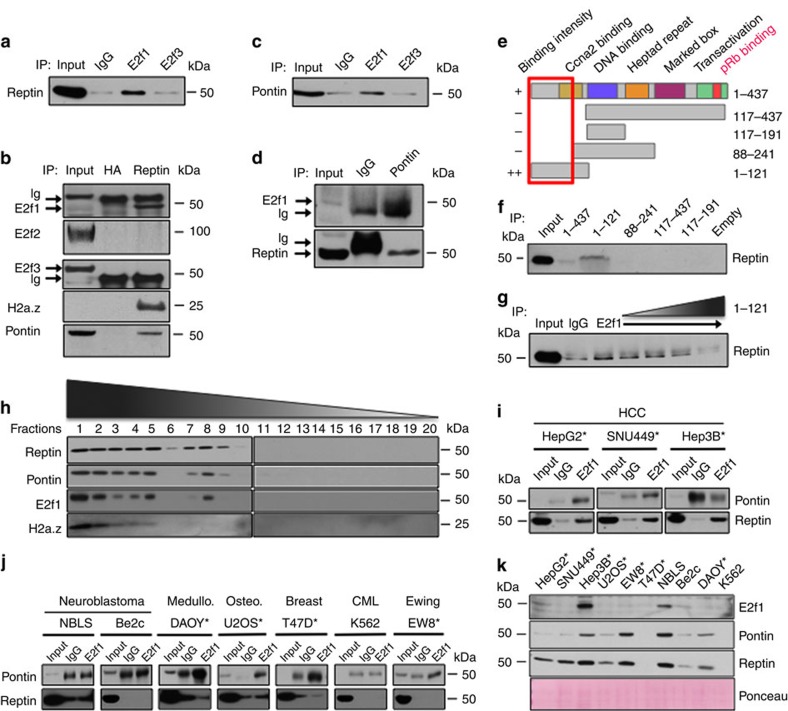
E2f1 recruits the Pontin/Reptin complex in TKO HCC. (**a**) Immunoprecipitation (IP) for IgG, E2f1 and E2f3 in TKO HCC cells. Endogenous E2f1 and E2f3 were pulled-down and the presence of Reptin was detected in the pulled-down fractions by immunoblotting. (**b**) IP for HA and Reptin in TKO HCC cells. Endogenous Reptin was pulled-down and the presence of E2f1, E2f2, E2f3, Pontin and H2a.z was detected in the pulled-down fractions by immunoblotting. H2a.z could only be detected upon IP/western blot. (**c**) IP for IgG, E2f1 and E2f3 in TKO HCC cells. Endogenous E2f1 and E2f3 were pulled-down and the presence of Pontin was detected in the pulled-down fractions by immunoblotting. (**d**) IP for IgG and Pontin in TKO HCC cells. Endogenous Pontin was pulled-down and the presence of E2f1 and Reptin was detected in the pulled-down fractions by immunoblotting. (**e**–**g**) GST constructs encompassing domains of human E2f1 were generated (**e**) and their interaction with endogenous Reptin from TKO HCC cells was determined by immunoblotting (**f**). (**g**) The endogenous IP described in **a** was repeated and increasing amounts of GST-E2f1-1-121 were added to test its capacity to displace the E2f1/Reptin interaction. The presence of endogenous Reptin in the E2f1 pulled-down fraction was detected by immunoblotting. The E2f1 antibody used in this experiment (C-20) recognizes the C-terminal domain of E2f1. (**h**) Nuclear extracts from TKO HCC cells were deposited at the top of a gradient and allowed to migrate through the gradient by ultracentrifugation. Twenty fractions were collected from the gradient and aliquots were run on a polyacrylamide gel to detect the migration pattern of Pontin, Reptin, E2f1 and H2a.z by immunoblotting. (**i**,**j**) Detection by IP of the interaction between E2f1 and Pontin/Reptin in human cancer cell lines: HepG2, Hep3B and SNU4499 (hHCC), NBLS and Be2c (neuroblastoma), DAOY (medulloblastoma), U2OS (osteosarcoma), T47D (breast cancer), K562 (chronic myeloid leukemia-CML) and EW8 (Ewing sarcoma). Asterisk marks cell lines where Pontin and/or Reptin interacts with E2f1. (**k**) E2f1, Pontin and Reptin expression levels in the different cell lines used in **i**,**j** as detected by immunoblotting. Normalization was assessed by Ponceau staining to ensure equivalent loading. *P<0.05; **P<0.01; ***P<0.001.

To confirm that E2f1 interacts with Pontin and Reptin, we performed immunoprecipitation (IP) and reverse-IP for endogenous proteins in TKO HCC cells. These assays showed that E2f1, but not E2f2 or E2f3, interacts with Pontin and Reptin in TKO HCC (Fig. 4a–d, see Supplementary Fig. 7a,b for the uncropped autoradiogram of Fig. 4a,c, respectively). To identify the domain of E2f1 involved in this interaction, we performed glutathione *S*-transferase (GST)-fusion pull-down assays and found that E2f1 recruits Reptin through residues 1–88 of its N-terminal domain (Fig. 4e–g, see Supplementary Fig. 7c for the uncropped autoradiogram of Fig. 4g). Using a similar GST pull-down assay, we could not pull-down Pontin with GST–E2f1. The E2f1 N-domain is not required for DNA binding and is poorly conserved within the E2f family^25^ (despite being very well conserved between mouse and human), thereby supporting the specificity of Pontin and Reptin recruitment by E2f1.

To determine the fraction of E2f1 that interacts with Pontin/Reptin, we performed a gradient assay with nuclear extracts from TKO HCC cells. The 20 collected fractions were run on a polyacrylamide gel and the presence of Pontin, Reptin, E2f1 and H2a.z was detected by immunoblotting. This assay showed that Pontin and Reptin co-migrate in two distinct patterns (lanes 1 to 5 and 7 to 9; Fig. 4h, see Supplementary Fig. 8 for the uncropped autoradiograms). Interestingly, E2f1 co-migrated with both patterns of Pontin/Reptin complex but could not be detected in the lighter fractions, indirectly suggesting that E2f1 is mostly found in complex with Pontin and Reptin, rather than alone, in TKO HCC. Finally, H2a.z could only be detected in the heavier fractions, again indirectly suggesting that H2a.z is only associated with the heavier forms of the Pontin/Reptin complex.

To determine whether the recruitment of Pontin/Reptin by E2f1 also occurs in the context of human disease, we repeated the pull-down fraction of E2f1 in three different human HCC (hHCC) cell lines. We found that Reptin was present in the E2f1 pull-down fraction in the three cell lines tested, whereas Pontin was detected in the E2f1 pull-down fractions in HepG2 and SNU449 cells, but not in Hep3B cells (Fig. 4i). Using a panel of human cancer cell lines of different origins (Fig. 4j), we found that E2f1 co-precipitated with Pontin in several lines, indicating that recruitment of homo- or hetero-Pontin/Reptin complexes by E2f1 is a common, albeit not ubiquitous, phenomenon in cancer. Detection of Pontin, Reptin and E2f1 in protein extracts of all cell lines used in Fig. 4i,j failed to show any correlation between the level of protein expression and the formation of Pontin/Reptin/E2f1 complex, suggesting that context-dependent events must occur to bring these proteins together (Fig. 4k).

### Pontin/Reptin open the chromatin at E2f target genes

**Figure 5:**
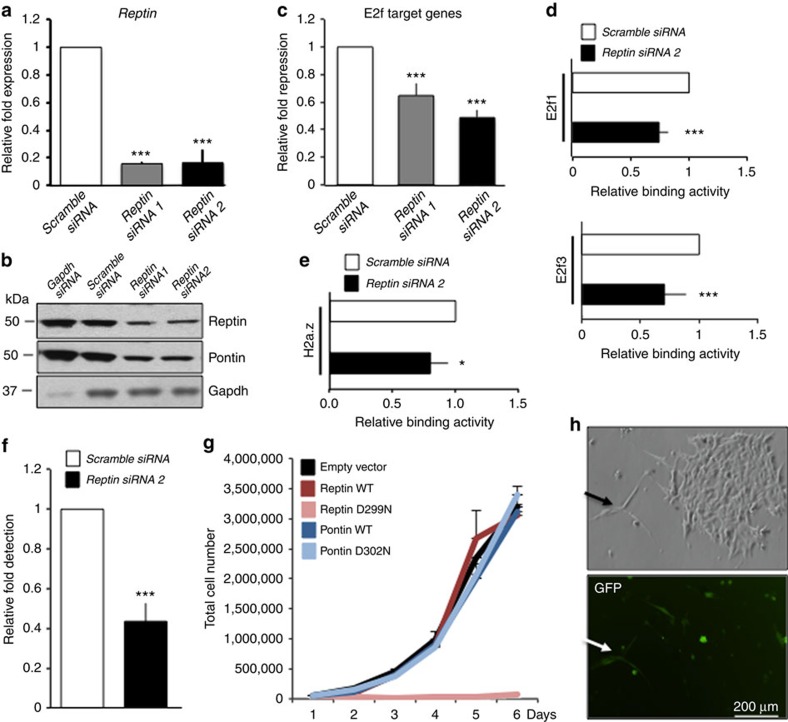
Critical role for the chromatin remodelling functions of Reptin in TKO HCC. (**a**) Intensity of *Reptin* silencing in TKO HCC cells upon their transfection with either *Scramble*, *Reptin1* or *Reptin2* si*RNAs*, as determined by quantitative PCR (qPCR; *n*=3). (**b**) Expression of Reptin (upper panel), Pontin (middle panel) and Gapdh (lower panel) in TKO HCC cells transfected with either *Scramble*, *Gapdh* or *Reptin1* or *Reptin2* si*RNAs*, as detected by immunoblotting (*n*=3). (**c**) Average expression level of the panel of E2f target genes (see Fig. 2b for genes list) upon transfection of TKO HCC cells with *Scramble* (white), *Reptin1* (grey) and *Reptin2 siRNAs* (black), as determined by reverse transcription–quantitative PCR analysis (*n*=3). (**d**,**e**) ChIP assay to detect the enrichment for E2f1, E2f3 (**d**) and H2a.z (**e**) at the promoter region of the panel of E2f target genes upon transfection of TKO HCC cells with either *Scramble* (white) or *Reptin2* (black) *siRNAs*, as determined by qPCR analysis (*n*=3). (**f**) Decreased DNAseI hypersensitivity upon *Reptin* silencing in TKO HCC cells. Accessibility of promoter regions from *cyclina2*, *mcm3*, *mcm6*, *glut4*, *gsk3b* and *pkm2* were assessed by qPCR, with non-promoter regions of *gapdh* as an internal control (*n*=3). (**g**) Growth curve of TKO HCC cells infected with the MigR1-IRES-GFP retrovirus either empty or expressing the wild-type (WT) and point mutant forms of human Pontin and Reptin. 50,000 cells were plated in six wells in triplicates and counted every day. This experiment has been performed three times independently and the panel displays the data from one representative experiment. (**h**) Representative image of cells expressing Reptin D299N point mutant. Cells expressing Reptin D299N (as identified by GFP^+^ expression, arrow) display a cellular crisis-like morphology and do not form colonies. They are outcompeted in the dish by rare GFP^−^ cells that display normal morphology and growth behaviour (colony on the right side of the picture). Normal morphology was also observed for all GFP^+^ cells expressing WT Pontin, WT Reptin and point mutant Pontin. Error bars represent standard deviation *P<0.05; **P<0.01; ***P<0.001.

To determine the consequence of Pontin/Reptin recruitment by E2f1 for E2f transcriptional response in TKO HCC, we silenced Reptin with two different short interfering RNAs (siRNAs) in TKO HCC cells. *Reptin* knock-down (Fig. 5a) led to decreased expression of both Reptin (63% decrease for Reptin siRNA1 and 61% decrease for Reptin siRNA1) and Pontin (52% decrease for Reptin siRNA1 and 53% decrease for Reptin siRNA1) proteins (Fig. 5b). This phenomenon has been described previously^26^ and indirectly suggests that Pontin/Reptin are mostly present as heteromeric complexes in TKO HCC. Importantly, *Reptin* knock-down repressed the expression of a panel of E2f target genes (Fig. 5c and Supplementary Fig. 4a) to ∼2-fold, which is similar to the repression observed upon expression of Rb-7LP in TKO HCC cells (Fig. 1d and Supplementary Fig. 4b) and correlate with the 2-fold amplification observed from early to late lesions (Fig. 2c–e). In addition, *Reptin* knock-down decreased the binding of both E2f1 and E2f3 to the regulatory regions of the panel of E2f target genes (Fig. 5d and Supplementary Fig. 4c), indicating that Pontin/Reptin recruitment by E2f1 increases the binding of E2f factors to E2f target genes and amplifies their transactivation. *Reptin* knock-down also decreased the presence of H2a.z, suggesting that Pontin/Reptin recruitment may alter the conformation of chromatin at the regulatory regions of E2f target genes in TKO HCC. To test this hypothesis, we performed a DNAseI hypersensitivity assay in TKO HCC cells upon *Reptin* knock-down and found that the partial silencing of *Reptin* expression led to decreased accessibility of the regulatory regions of E2F target genes (Fig. 5f). Overexpression of wild-type Pontin and Reptin did not alter the proliferative capacity of TKO HCC cells *in vitro*. In contrast, expression of a point mutant form of Reptin (D299N, which ablates the chromatin remodelling capacity of Reptin)^27^, but not Pontin, abolished the proliferative capacity and altered the morphology of TKO HCC cells (Fig. 5g,h). Finally, partial knock-down of E2f1 led to a modest reduction of E2f target gene expression but failed to modify the proliferative capacity of TKO HCC cells, in line with previous reports (Supplementary Fig. 4d,e). Collectively, these data indicate that the recruitment of Pontin/Reptin complexes by E2f1 opens up chromatin conformation at E2f target genes and amplifies their transcription by E2f factors.

### Progressive formation of the E2f1-Pontin/Reptin complex

**Figure 6:**
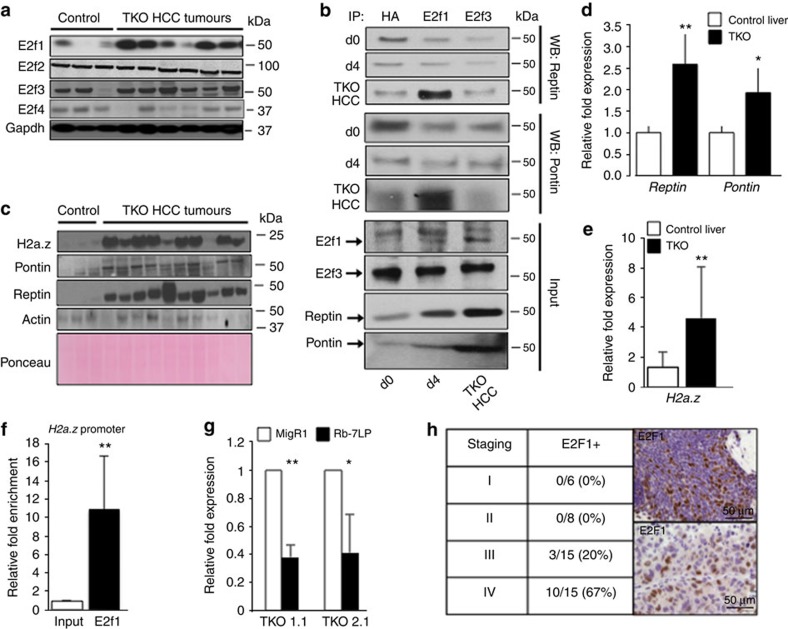
Progressive recruitment of Pontin/Reptin by E2f1 in TKO HCC. (**a**) E2f1, E2f2, E2f3, E2f4 and Gapdh (normalization) expression levels in control liver and TKO HCC, as detected by immunoblotting. (**b**) IP for HA, E2f1 and E2f3 in primary liver extracts from cTKO (d0), *Rosa26-CreER*^*T2*^ *TKO* 4 days after Tamoxifen treatment (d4) and TKO HCC. Reptin (upper panels) and Pontin (middle panels) were detected in the pull-down fractions at each time point by immunoblotting. Expression levels of E2f1, E2f3, Reptin and Pontin were detected at the different time points in the corresponding input fractions by immunoblotting (lower panels). (**c**) H2a.z, Pontin, Reptin and Actin protein expression in control liver and TKO HCC, as detected by immunoblotting. Ponceau staining is used for normalization purpose. (**d**) mRNA expression for *Reptin* and *Pontin* in control liver (*n*=5) and TKO HCC (*n*=9), as detected by qPCR. (**e**) mRNA expression for *H2a.z* in control liver (*n*=5) and TKO HCC (*n*=9), as detected by qPCR. (**f**) Binding of E2f1 to the promoter region of *H2a.z* (following the identification of an E2f-binding site in the promoter of *H2a.z*), as shown by ChIP assay in TKO HCC cells (*n*=3). (**g**) Repression of *H2a.z* expression in two clones of TKO HCC cells (2.1 and 1.1) upon infection with retroviruses either control (MigR1) or expressing the Rb-7LP protein (*n*=3). (**h**) Left: Frequency of E2F1 expression in tissue microarray (TMA) encompassing human HCC cases from stages I–IV. Right: Representative E2F1 staining of two independent HCC cases classified as stage IV HCC. Error bars represent standard deviation *P <0.05; **P <0.01; ***P<0.001. WB, western blot.

Data from Figs 1 and 2 suggest that the amplification of E2f transcriptional response is a progressive phenomenon in TKO HCC. To identify the mechanism that underlies the progressive nature of this phenomenon, we first determined the evolution of E2f factors expression in TKO HCC. Although E2f2, E2f3 and E2f4 protein expression levels are mostly unaltered, we found that E2f1 expression increases in TKO HCC compared with control livers (Fig. 6a; an average of 2.61-fold induction for E2f1 compared with controls, 1.07- for E2f2, 1.3- for E2f3 and 0.84-fold induction for E2f4). In line with this result, E2f1 is only able to recruit Pontin and Reptin in TKO HCC but not upon acute Rb family inactivation, which correlates with the progressive accumulation of E2f1, as well as Pontin and Reptin (Fig. 6b). In addition to E2f1, we found that Pontin, Reptin and H2a.z also display higher expression in TKO HCC compared with control livers (Fig. 6c–e, see Supplementary Fig. 9a for the uncropped autoradiograms and the Ponceau staining of Fig. 6c; an average of 11.44-fold for H2a.z compared with controls, 3.39-fold for Pontin and 4.02-fold for Reptin) and our data identified *H2a.z* as a new E2f target gene (Fig. 6f,g). To correlate these findings with the human disease, we performed staining of Tissue Microarray with anti-E2F1 antibody and found that E2F1 is restricted to the latter stages of hHCC, indirectly indicating that E2F1-Pontin/Reptin complex can only be observed in the advanced stages of the hHCC (Fig. 6h). Together, these data suggest that the progressive nature of E2f transcriptional response amplification in TKO HCC results from the combined accumulation of E2f1, Pontin, Reptin and H2a.z during TKO HCC progression, which recapitulates the expression of these factors in the hHCC. However, data from Fig. 4k suggest that the global mechanism of Pontin/Reptin/E2f1 complex formation in cancer is likely more complex.

### E2f-binding affinity dictates the kinetic of activation

**Figure 7:**
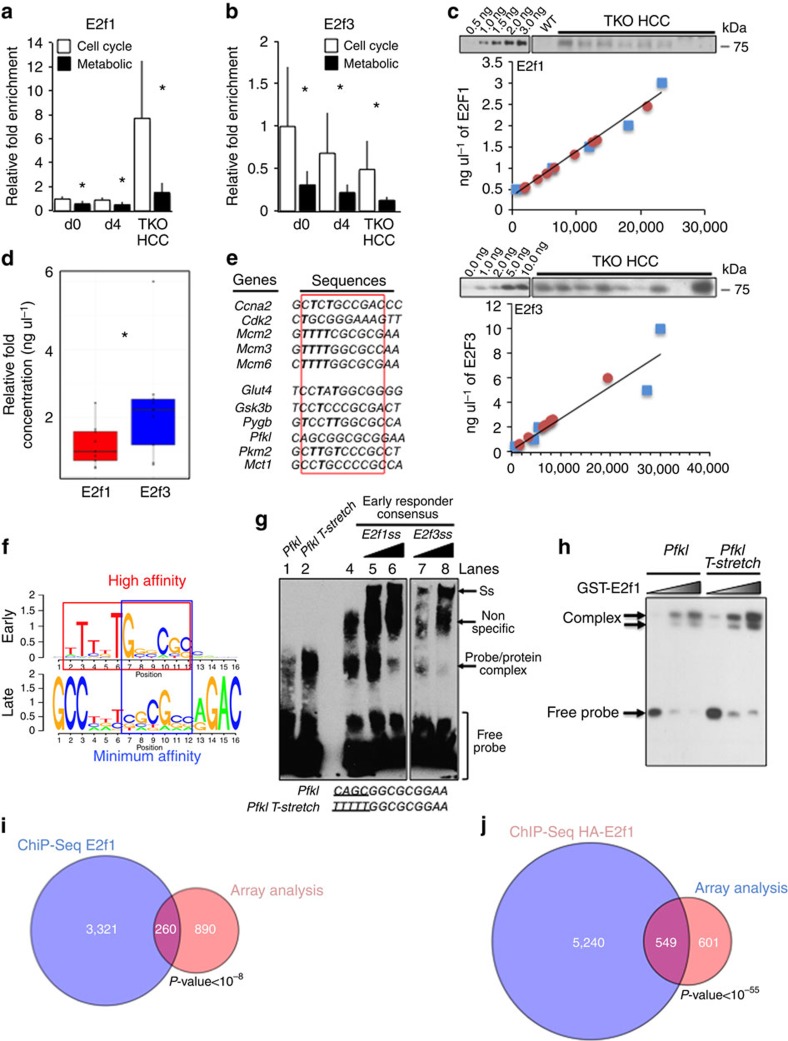
Distinct classes of E2f target genes based on E2f affinity for their promoter regions. (**a**,**b**) ChIP assays for E2f1 (**a**) and E2f3 (**b**) to detect their enrichment at the promoter regions of representative cell cycle or metabolic target genes (see Fig. 2b for gene lists) at different time points upon Rb family loss, as determined by quantitative PCR (*n*=3). (**c**,**d**) Quantification of E2f1 and E2f3 proteins expressed in TKO HCC. Protein extracts from TKO HCC tumours were run on a polyacrylamide gel in parallel with increasing amount of recombinant E2f1 (**c**, upper panel) and E2f3 (**c**, lower panel) proteins. Expression of E2f1 and E2f3 was quantified using the standard curve established with recombinant proteins. The average expression is displayed in **d** for better comparison. The box represents the data included within the 25th to 75th percentile. The horizontal bar represents the average. (**e**) Alignment of ChIP validated E2f-binding sites from cell cycle and metabolic target genes. Canonical E2f-binding site is found within the red rectangle and thymine residues are in bold. (**f**) Identification of consensus binding sites for early and late sets of E2f target genes. The consensus site for the early genes was obtained by collapsing the sequences of the E2f-binding sites identified in the set of 296 genes (see Fig. 2c for details). The consensus site for the late genes was obtained by collapsing the sequences of the E2f-binding sites located in the promoter region of the metabolic target genes and the Notch signalling genes^11^. The red box identifies the high-affinity binding site, which includes the T-stretch. The blue box identifies the minimal sequence, 5′-SSCGC-3′, required for low-affinity binding. (**g**) Gel shift assay to determine E2f1- and E2f3-binding affinity to probes containing either the early *consensus* sequence, the *Pfkl* sequence and a T-stretch mutated *Pfkl* sequence. Complexes were supershifted with increasing amount of E2f1 or E2f3 antibodies. Ss: supershift. Free probe is displayed at the bottom of the panel. (**h**) Gel shift assay with increasing amount of recombinant GST–E2f1 incubated with constant amount of the *Pfkl* and *Pfkl T-stretch* probes. (**i**,**j**) Intersection of genes transactivated in TKO HCC with gene sets identified in the E2f1 ChIP-Seq experiment performed in TKO HCC (**i**) and in MCF7 cells stably expressing HA-E2f1 by the Farnham group (**j**). *P*-value obtained by hypergeometric test. Error bars represent standard deviation *P <0.05; **P <0.01; ***P<0.001.

Amplification of E2f transcriptional response by Pontin/Reptin recruitment does not elucidate the mechanism that underlies the existence of distinct waves of E2f target genes during TKO HCC progression. ChIP assay with E2f1 and E2f3 upon acute Rb family deletion in primary cTKO liver (d0, d4) and in TKO HCC showed an inverse evolution of E2f1- and E2f3-binding intensity during TKO HCC progression (Fig. 7a,b). The relative ratio of E2f1 (average of 1.47 ng μl^−1^) versus E2f3 (average of 2.27 ng μl^−1^) protein abundance in TKO HCC correlates with the twofold reduction of E2f3 binding over time and E2f1 accumulation (Fig. 7c,d). Importantly, we found that E2f1 and E2f3 consistently bound with higher affinity to cell cycle target genes compared with metabolic target genes at any time of TKO HCC progression (Fig. 7a,b; see Fig. 2b for the list of the 11 target genes). Based on these data, we hypothesized that the sequence of the E2f-binding site located in the promoter region of E2f target genes plays a crucial role in dictating E2f-binding activity and consequently the kinetic of activation by E2f factors during TKO HCC progression. Of note, the 5′-TTTSSCGC-3′ sequence is considered the canonical binding sequence for E2f, but work from the Farnham group showed that the 5′-SSCGC-3′ sequence is sufficient for E2f1 binding, with a minimal role for neighbouring sequences located around the E2f-binding site^25^. Accordingly, alignment of literature- and ChIP-validated E2f-binding sites for genes from both early (296 cell cycle genes as identified in Fig. 2c) and late (metabolic and Notch pathway target genes) waves led to the establishment of a consensus sequence for both groups. Although the 5′-SSCGC-3′ sequence was overall conserved in both groups, only genes from the early wave display a 5′ T(thymine)-stretch adjacent to this minimal binding site (Fig. 7e,f). To test whether the T-stretch is critical to determine E2f-binding affinity to target genes, we performed gel shift assay with biotin-labelled probes containing the early consensus (as identified in Fig. 7f) and the *Pfkl* (which does not contain any thymine residue) sequences. We found that the early consensus probe bound to E2f1 and E2f3 (lanes 4–8 for supershift (Ss) with increasing amount of anti-E2f1 and-E2f3 antibodies) with more intensity than the *Pfkl* probe (Fig. 7g, compare lanes 1 and 4). However, the inclusion of a T-stretch in the *Pfk*l probe increased E2f binding to a level similar to the early consensus probe (compare lanes 1–2 and 4). Gel shift performed in a *in vitro* context with increasing amount of GST–E2f1 and identical quantity of Pfkl and Pfkl T-stretch probes showed that E2f1 display higher affinity for the latter probes (Fig. 7h and Supplementary Fig. 5a, see Supplementary Fig. 9c for the uncropped autoradiogram). These results show that the presence of the T-stretch is a unique feature of early E2f target genes and increases the affinity of E2f for binding to target genes, suggesting that the sequence of the E2f-binding site present in the regulatory region of E2f target genes underlies the kinetics of E2f target genes activation upon sustained E2f activity.

Finally, we analysed the intersection of genes bound by E2f1 (obtained from the ChIP-Seq experiment described in Fig. 3c,d) and genes transactivated in TKO HCC. Albeit significant (*P-*value<10^−8^), the overlap was fairly limited, with 260 genes present at the intersection of both data set (Fig. 7i and Supplementary Data 6). In contrast, analysis of the genes transactivated in TKO HCC and the genes bound by haemagglutinin (HA)-tagged exogenous E2f1 in breast cancer cells^25^ identified 549 genes at the intersection of both data sets (*P-*value<10^−55^; Fig. 7j), suggesting that ChIP-Seq experiments performed by pulling-down endogenous proteins may only detect high-affinity binding target genes and ignore a significant fraction of target genes bound by E2f1 with low affinity.

## Discussion

**Figure 8:**
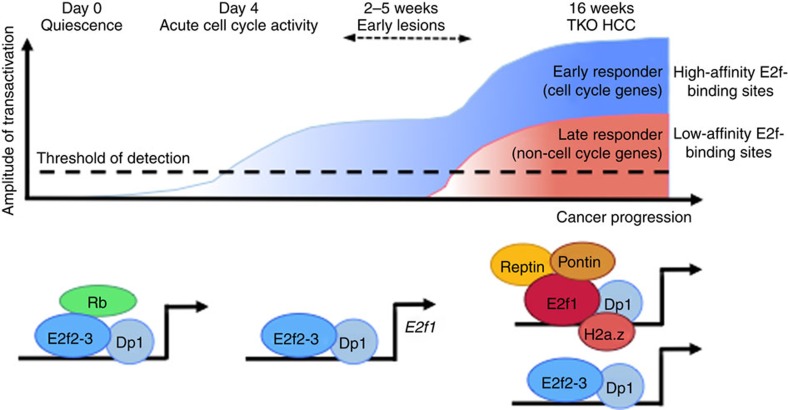
Progressive amplification of E2f response during cancer progression. Model for the evolution of E2f transcriptional output in TKO HCC. Upon Rb family loss, TKO liver cells enter cell cycle activity. Early lesions composed of proliferative Sca1^+^ progenitor-like cells can be detected after 2–5 weeks and gradually turn into late lesions macroscopically visible by ∼4 months of age (TKO HCC). In quiescent cells, Rb family represses the transcriptional activity of E2f and the dimerization partner Dp1. Upon acute Rb family loss, E2f2 and E2f3 activate the transcription of cell cycle genes and *E2f1*. During TKO HCC progression, E2f1 gradually binds to the promoter region of E2f target genes and recruits Pontin and Reptin to incorporate H2a.z and amplify E2f transcriptional response. This mechanism leads to increased transactivation of cell cycle target genes (early genes displaying high-affinity E2f-binding sites in their promoter regions) and activation of non-cell-cycle target genes (late genes displaying low-affinity E2f-binding sites in their promoter regions), including genes that regulate the Warburg effect, Notch signalling and most likely other oncogenic features.

Our data have identified an epigenetic mechanism recruited by E2f1 to amplify E2f transcriptional response during the progression of a pre-clinical model of HCC. We show that the net outcome of this mechanism is the enhanced transactivation of cell cycle genes and the *de novo* transactivation of novel E2f target genes that display low-affinity E2f-binding sites in their regulatory regions, such as genes regulating glucose metabolism and the Warburg effect (Fig. 8).

In the current model of tumour evolution, a succession of independent genetic and epigenetic events drives discrete changes in the transcriptional landscape of the tumour, leading to the progressive acquisition of new oncogenic features that support tumour development^28^,^29^. Our data introduce an alternative model (although not mutually exclusive) wherein the E2f transcriptional response gradually evolves over time following the amplification of E2f transcriptional activity. These results suggest that E2f transcriptional response upon transient E2f activation is limited to high binding affinity target genes that regulate the progression through cell cycle. In contrast, E2f transcriptional response upon sustained E2f activity expands to many additional target genes that harbour low-affinity E2f-binding sites in their regulatory regions. Here, we have identified a set of these low-affinity target genes that regulate the Warburg effect. Therefore, our data establish E2f as a central hub in the regulation of proliferation and energy metabolism in cancer cells. Our previous work identified additional E2f target genes with non-cell-cycle functions in the context of Rb family deficiency, such as the Notch pathway genes for liver cancer^11^ and *Foxn1* for thymus development^30^. Still, it is likely that these target genes only represent a small fraction of low-affinity E2f-binding genes. This prediction is in agreement with our ChIP-Seq results and ChIP-Seq results generated by the Farnham group, which led to the identification of more than 10,000 binding sites (corresponding to 5,789 genes) for E2f1 in breast cancer cells, the majority of which only harbours the 5′-CGCGC-3′ binding site^25^. However, the fact that E2f factors have low binding affinity for these target genes suggest that their detection in genome-wide sequencing approaches may be technically challenging. This possibility is further supported by the comparison of our ChIP-Seq analysis, performed with endogenous E2f1 protein, and ChIP-Seq performed previously with overexpressed tagged E2f1 (Fig. 7i,j). Improvement of ChIP techniques is needed to identify low-affinity E2f target genes but it is likely that their functions expand well beyond the regulation of cell cycle activity. Finally, these ChIP-Seq data identify many genes bound, but not transactivated, by E2f1 in TKO HCC (Fig. 7i, 3,321 hits), which suggests that a permissive chromatin environment or the presence of additional co-factors is necessary for the efficient transactivation of genes bound by E2f factors.

Previous reports have shown that the recruitment of Pontin/Reptin complex increases the transcriptional response of Myc^31^ and Oct4 (ref. 32), suggesting that this complex modulates transcriptional activity in a number of different cellular and molecular contexts. Mechanistically, Pontin and Reptin are related to a family of AAA^+^ ATPase helicases and they display multiple functions such as the regulation of transcription, DNA repair and chromatin remodelling protein^24^. Results shown in Fig. 5 indicate that the recruitment of Pontin/Reptin by E2f1 opens chromatin at E2f target genes (as evidenced by the decreased presence of H2a.z and the reduced DNAseI sensitivity upon *Reptin* silencing in TKO HCC cells), which impacts the capacity of E2f factors to bind to their target genes. Indeed, our data in Fig. 7a–d show that the regulatory regions of E2f target genes are occupied by several E2f factors and the consequence of Pontin/Reptin activity on chromatin conformation may explain why E2f3 binding is also impacted by *Reptin* knock-down, although it does not physically interact. However, this model requires further experimental validation and it is likely that the mechanism of transcriptional amplification by Pontin/Reptin is more complex. Last, our data indicate that only the chromatin remodelling activity of Reptin is required in the TKO HCC setting. In contrast, Pontin interacts with Reptin and E2f1 but its chromatin remodelling activity is not required in TKO HCC (Fig. 5g). As shown in Fig. 5b and demonstrated by others, Reptin and Pontin depend on each other for their stability. In the context of TKO HCC, we therefore hypothesize that the role of Pontin in the E2f1-Pontin/Reptin association may be limited to scaffolding functions that stabilize Reptin expression and enable Reptin-mediated chromatin remodelling activity. However, more mechanistic studies will be required to understand this phenomenon, which is likely to be cell-type dependent.

Our unbiased MS approach has led to the identification of many interacting partners for E2f1 and E2f3. Surprisingly, the only common interactor for both E2f proteins is Dp1, which is required for their transcriptional activity. The DNA-binding domain is highly conserved among E2f members^1^ and genetic experiments and transcriptional profiling have indicated strongly overlapping list of target genes for activator E2fs^1^. Our data suggest that the specificity of activator E2f functions may not reside in the identity of bound target genes but rather in the capacity of the different activator E2fs to recruit co-factors to modulate the transactivation of otherwise common target genes in a context-dependent manner.

Non-DNA-binding regions are poorly conserved among E2f factors and it is therefore not surprising that E2f1 recruits Pontin/Reptin through its N-terminal domain. The N-domain of E2f1, which is highly conserved across species, is also necessary to recruit know interactors of E2f1 such as CyclinA2. The fact that E2f1 appears to recruit so many interactors, including several chromatin regulators such as Pontin/Reptin, Mll2 and Swi/Snf complex, raises multiple questions regarding these interactions: do they occur simultaneously, sequentially or in a competitive manner? And are post-translational modifications required for the recruitment of these interactors? Although work from others has already shed light on the recruitment of co-factors by E2f1, our data indicate that this process is likely very complex and more work is need to fully understand its mechanism and transcriptional consequence.

The evolution of E2f transcriptional response in TKO HCC correlates with the evolution of the transcriptional landscape during hHCC progression, including the expression of key markers of hHCC progression such as CD34 and *Igfals* (Supplementary Fig. 6a,b)^33^. Increased expression of E2f1 is a common feature of several types of cancers such as melanoma and HCC^10^. Although the mechanism of action is poorly understood, it appears that E2f1 activity plays a critical role in the progression of these cancers at an established stage. In parallel, overexpression of Pontin and Reptin is also a frequent feature of many types of cancer, including HCC^22^,^34^. Work by the Rosenbaum group has shown that repression of Pontin/Reptin expression or chemical inhibition of their ATPase activity is sufficient to inhibit the proliferation of HCC cells *in vitro* and in xenograft transplants^27^,^35^,^36^. Although Pontin and Reptin have other binding partners that are relevant for cancer progression such as β-catenin and Myc^31^,^37^, our results, combined with the frequent aberrant E2f activity in cancer, suggest that their recruitment by E2f1 may be a common mechanism to drive cancer progression. Impairing the enzymatic activity of Pontin/Reptin *in vivo* is likely to induce strong adverse effects (supported by the early lethality of Pontin-deficient mice). However, impairing the recruitment of Pontin/Reptin by E2f1 may prove a valuable alternative strategy. In the future, it will be important to determine the mechanism of Pontin/Reptin recruitment by E2f1 in order to establish the therapeutic relevance of targeting this interaction.

## Methods

### Mice

Rb-family cTKO mice^38^ were maintained on a mixed 129Sv/J;C57/BL6 background. For intrasplenic injections, mice (male and female, between two and four months of age) were anaesthetized and surgically opened on the upper left flank and adenovirus was injected into the spleen. In *Rosa26-CreER*^*T2*^ *TKO* mice, Cre expression was induced by intraperitoneal injection of 1 mg of Tamoxifen (Sigma) in corn oil for 5 consecutive days. Mice were housed in the CHOP barrier facility. All experiments with mice were approved by the Children's Hospital of Philadelphia Institutional Animal Care and Use Committee (CHOP IACUC) (protocol #969).

### Cell culture and infections

TKO HCC cells were cultured in DMEM with sodium pyruvate, L-glutamine and phenol red (Mediatech), supplemented with 10% fetal calf serum and 1% penicillin–streptomycin–glutamine (Invitrogen). Rb-family TKO1.1 and 1.2 cell lines were derived from independent liver tumours in Rb-family TKO mice^11^. Experiments have been performed in both cell lines with similar results and they are collectively referred to as TKO HCC cells. A super-repressor form of Rb containing the Rb large-pocket domain (Rb-7LP^39^, a generous gift from Eric Knudsen) was inserted into the *Xho*I site of MigR1-IRES-GFP retroviral (generously provided by Warren Pear, University of Pennsylvania). cDNA of human Pontin and Reptin, either wild-type or their point mutant versions (D302N and D299N, respectively; a kind gift of Jean Rosenbaum, Bordeaux, France) were also subcloned in the MigR1-IRES-GFP retroviral vector. An empty MigR1-IRES-GFP vector was used as a mock infection control. Retroviral vectors were transfected into PlatE cells by calcium phosphate transfection as previously described^40^. TKO cells were infected with media from transfected PlatE cells and collected after 24, 48 or 72 h. Three hairpins corresponding to Scramble or targeting *E2f1* mRNA were cloned in pSicoR-GFP. Lentiviral supernatant was produced in 293T cells cultured in DMEM. Silencer Select siRNAs (*Gapdh*, *Scramble*, *Reptin 1&2* siRNA, Invitrogen) were transfected into TKO HCC cells with Lipofectamine 2000 (Invitrogen). Block-iT FITC-tagged oligos (Invitrogen) were used as transfection controls. Cell media were changed 4 h following infection. Cells were harvested after 48 or 72 h following transfection for ChIP analysis, RNA or protein extraction. The panel of human cancer cell lines was grown in DMEM or RPMI supplemented with 10% FBS and 1% penicillin–streptomycin–glutamine.

### Metabolic assessment

For growth assays, 50 × 10^5^ cells were seeded and grown in DMEM containing glucose (4.5 g l^−1^) or glucose-free DMEM supplemented with galactose (4.5 g l^−1^). Cells were counted manually on a haematocytometer for 5 days. For metabolite concentration assays, TKO HCC cells were infected with either empty or Rb7LP-MigR1-IRES-GFP. Cells were collected 24 h after infection and sorted for GFP expression on a MoFloXDP sorter (Beckman-Coulter). The GFP^+^ fraction was replated and cultured for 48 or 72 h. Media were collected from cell cultures and assayed using an YSI 7100 reader (equipment generously provided by Kathryn Wellen, University of Pennsylvania). Reactive oxygen species (ROS) production was assayed using MitoSOX Red Mitochondrial Superoxide Indicator (Invitrogen, M36008). TKO cells infected with Rb7LP-MigR1 or empty MigR1 were harvested after 48 or 72 h and stained with Mitosox according to the manufacturer's specifications. Cells were analysed on a C6 Accuri analyzer (BD). For paraffin sections, organs were fixed in 4% formaldehyde overnight at room temperature and transferred to 70% ethanol before processing and embedding. Periodic acid–Schiff base staining was performed according to the manufacturer's specifications (Sigma, 395B-1KT). Slides were counter stained with haematoxylin and eosin where indicated. Slides were imaged on an Olympus IX81 inverted microscope.

### Quantitative PCR

Total RNA was extracted from cell lines and primary tissue directly into either Trizol or Trizol LS for FACS-sorted cells (Life Technologies) and purified with the RNeasy Mini kit (Qiagen). Complimentary DNA was generated with the Protoscript First Strand cDNA Synthesis kit (New England Biolabs). Quantitative PCR was performed in duplicate with SYBR Green PCR Master Mix (Life Technologies) on the Viia7 Real-Time qPCR system (Life Technologies). Data were normalized using *actin* or *gapdh* as a reference gene. Primer sequences are available in Supplementary Table 1.

### ChIP assay and ChIP-Seq

Cell lines were collected into 0.5% SDS lysis buffer at a density of 50 × 10^6^ cells per ml and crosslinked in 2% formaldehyde for 10 min. Lysates were sonicated on a BioLogics Model 3000 Ultrasonic Homogenizer for 30 s at 40% power on ice to produce chromatin fragments of ∼400–700 bp. For ChIP experiments using primary liver tissue, livers were perfused *in situ* via the inferior vena cava with 30 ml of 2% formaldehyde over 5 min. Livers were removed, weighed, minced and homogenized in a Potter-Elvehjem hand-powered homogenizer. Homogenate was treated an additional 10 min in 2% formaldehyde to crosslink. Primary cells were washed, filtered through a 70-μm filter, lysed in buffer containing 1% SDS at a density of 0.1 g tissue per ml and sonicated for eight cycles of 30 s at 40% power followed by 30 s of rest. Cell lysates were pre-cleared with Protein A-conjugated Sepharose beads (Life Technologies) for 1 h at 4 °C. Pre-cleared lysates were then incubated overnight with 10 μg antibody at 4 °C. Antibodies used for IP are as follows: E2F1 (Santa Cruz, SC-193X), E2F3 (Santa Cruz, SC-878X), H2a.z (Abcam, ab4174), H3k27ac (Abcam, AB4729), H3k27me3 (Millipore, 07-449), H3k9ac (Abcam, AB10812), H3k9me3 (Abcam, AB8898), H3k4me3 (Abcam, AB8580), H3k4me1 (Abcam, AB8895), H4k20me3 (Abcam, AB9053), H3r17me2 (Abcam, AB8284), H3s10ph (Abcam, AB5176). Antibodies were pulled down with Protein A beads for 1 h at 4 °C. Beads were collected, washed and eluted. Eluates were treated with RNase and Proteinase K for 30 min at 37 °C then treated with NaCl and incubated overnight at 65 °C to reverse crosslinks. DNA was purified from eluates using phenol-chloroform isoamyl alcohol extraction. DNA fragments were analysed by quantitative PCR with SYBR Green reagents. Samples were normalized to input DNA. We could not perform ChIP assays for E2f2, because of the lack of a specific antibody available for this purpose.

To perform ChIP-Seq for H2a.z and E2f1, a similar procedure was achieved to isolate the genomic fraction. Genomic DNA samples (input and pull-downs) was quantified by bioanalyser and further processed by the genomic core facility at the University of Pennsylvania. Raw data are available in Supplementary Data 7 and 8. We used a list of genes previously identified as E2F1-bound genes in MCF7 cells by the Farnham group. To perform this experiment, this group generated stable transfectant of MCF7 cells expressing HA-E2F1-ER cDNA, where E2F1 is activated upon Tamoxifen treatment and E2F1 was pulled-down for ChIP-Seq using an anti-HA antibody.

### GST fusion pull-down assay

E2F1 GST-fusion proteins were produced in BL21 strain. Bacteria were grown to an optical density (*A*_595_) of 0.5, and then induced with 0.5 mM isopropyl-1-thio-β-D-galactopyranoside for 2 h before harvesting. Bacterial pellets were lysed in 30 ml of NENT buffer (250 mM NaCl, 1 mM EDTA, 20 mM Tris, pH 8, 1.5% Nonidet P-40) and sonicated three times for 15 s at 4 °C. Debris was removed via centrifugation. GST fusion proteins were purified by incubating the saved supernatant with 40 μl GST-sepharose beads for 2 h at 4 °C. Beads were washed two times in 1 ml of NENTM buffer (NENT, 0.5% milk) and once with TWB buffer (20 mM HEPES, pH 7.9, 60 mM NaCl, 2 mM dithiothreitol, 6 mM MgCl_2_, 8.2% glycerin, 0.1 mM EDTA). Fusion proteins were run on a 4–12% polyacrylamide gel then visualized for normalization with Coomassie blue. GST pull-down assay was performed by incubating 1 ml TKO cell lysates with an aliquot of GST bead-bound fusion protein in 200 μl of TWB buffer for 1 h. Beads were washed five times with 1 ml of NENTM buffer, loaded with 4 × LDS Sample Buffer w/.05% β-mercaptoethanol, then run on a 4–12% SDS–polyacrylamide gel for autoradiography.

### Lysates for MS and IP

Cell lines were harvested in HEPES Lysis Buffer (200 mM K-HEPES, pH 7.4, 1.1 M KOAc, 20 mM MgCl_2_, 1% Tween-20, 10 μM ZnCl_2_, 10 μM CaCl_2_, 500 mM NaCl, 2% Triton X-100, Protease Inhibitor Cocktail) at a density of 40 × 10^6^ cells per ml and were pre-cleared for nonspecific binding with Protein A-conjugated Sepharose beads for 1 h at 4 °C. Relevant antibodies were incubated with lysates for 1 h at 4 °C. 40 μl of Protein A-conjugated sepharose beads were added to lysates and incubated for an additional 1 h at 4 °C. Beads were washed three times in lysis buffer then unconjugated with 20 μl of 4 × LDS Sample Buffer w/.05% β-mercaptoethanol. Antibodies used were HA (Santa Cruz, SC-7392), E2F1 (Santa Cruz, SC-193), E2F3 (Santa Cruz, SC-878), Reptin (Sigma, 2E9-5) and Pontin 52 (Santa Cruz, SC-81360).

### Mass spectrometry

Each sample was run on an SDS–polyacrylamide gel electrophoresis, cut into eight even slices, further cut into 1 mm cubes reduced, alkylated and digested with trypsin. Tryptic digests were analysed by LC-MS/MS on a hybrid LTQ Orbitrap Elite mass spectrometer. All MS/MS samples were analysed using Sequest (Thermo Fisher Scientific), against a Uniprot *Mus musculus* complete proteome database (20130819, 51891 entries) appended with common contaminants. Scaffold (version Scaffold_4.3.4, Proteome Software Inc.) was used to validate MS/MS-based peptide and protein identifications.

### Protein biochemistry

To quantify the abundance of E2F1 and E2F3 proteins, we used the corresponding recombinant proteins (Origene) and loaded increasing amounts on a polyacrylamide gel, in parallel with protein extracts from TKO HCC. This strategy enabled the establishment of a standard curve that supports the quantification of E2F1 and E2F3 expression in TKO HCC. To perform a gradient, we loaded ultracentrifuge tubes with 5 ml of 20% Nycodenz and enabled the establishment of a gradient by a 30-min ultracentrifugation at 100,000 *g*. A measure of 100 μg of nuclear extract from TKO HCC cells were loaded on top of the gradient and allowed to migrate through the gradient by another round of ultracentrifugation. Twenty fractions of 250 μl were collected and 40 μl of each fraction was loaded on a polyacrylamide gel.

For immunoblotting, unconjugated lysis samples were run on a 4–12% polyacrylamide gel for 1.5 h at 150 V. Proteins were transferred to a nitrocellulose membrane, blocked in 5% milk for 1 h then incubated at 4 °C with primary antibody overnight. Membrane was washed in TBST three times, and then probed with an HRP-conjugated secondary antibody (anti-mouse, anti-rabbit, anti-goat) for 1 h. Membrane was washed again three times in TBST. Amplified signal was detected via ECL autoradiography. Antibody used: E2F1 (sc-193, 1/1,000), E2F2 (sc-833, 1/1,000), E2F3 (sc-893, 1/1,000), E2F4 (sc-1082, 1/500)), Actin (Cell Signaling, 4970, 1/10,000), GAPDH (sc-25778, 1/5,000), Pontin (Sigma, SAB4200194, 1/500), Reptin (Sigma, SAB1200115, 1/1,000), H2a.z (ab4174, 1/1,000), Glut4 (ab33780, 1/1,000), Gsk3b (Cell Signaling, 56765, 1/1,000). Representative uncropped immunoblotting images are available in the Supplementary Figs 7–9.

For immunohistochemistry, mouse HCC specimens were fixed in 10% buffered formalin and embedded in paraffin. Standard deparaffinization, rehydration and heat-induced epitope retrieval were performed. CD34 rabbit monoclonal antibody (1:500, Abcam, cat.81289) was used as primary antibody, and the reactions were visualized using the Vectastain ABC and DAB kit (Vector Laboratory). For hHCC staining, tissue microarray were obtained from Biomax (HLiv-HCC060CD-01) and processed as described above for antigen retrieval. Anti-E2F1 antibody (Abcam, ab4070, 1/100) was used to detect E2F1 expression.

### Gel shift assay

TKO HCC cells were harvested in 10 ml PBS at a density of 20 × 10^6^ cells per ml. Cells were washed then resuspended in 200 μl of Cytoplasmic Lysis Buffer (10 mM HEPES, pH 7.9, 10 mM KCl, 2 mM MgCl_2_, 0.1 mM EDTA, 0.1% NP-40, Protease Inhibitor Cocktail) and incubated on ice for 5 min. Lysates were spun down and pellet was washed twice in Wash Buffer (10 mM HEPES, 20 mM KCl, 2 mM MgCl_2_, 0.1 mM EDTA, Protease Inhibitor Cocktail). The pellet was then resuspended in twice its volume worth of Nuclear Extraction Buffer (20 mM HEPES, pH 7.9, 1.5 mM MgCl_2_, 0.2 mM EDTA, 0.63 mM NaCl, 25% glycerol, Protease Inhibitor Cocktail) and incubated on ice for 30 min. Lysates were spun down and supernatant collected as the nuclear extract. For DNA:protein binding reactions, the following reagents were mixed and incubated at room temperature for 20 min: NanoPure H_2_O, 7 μl Binding Buffer D (20 mM HEPES, 10 mM KCl, 0.2 mM EDTA, 10% glycerol, 5 mM dithiothreitol freshly added), 50 ng Poly dI:dC, 0.15% NP-40, 1 μg BSA, 5 μg nuclear extract and 20 fmol biotin-labelled double-stranded DNA oligos. For the supershift assay, 5 μg of polyclonal antibodies were added to the mix and pre-incubated for an additional 20 min before adding the biotin-labelled double-stranded DNA oligos. Total reaction volume was 20 μl for all samples. 5 μl of 5 × Loading Buffer (Thermo 20148K) were added to samples, which were then loaded and run on a pre-electrophoresed 4% polyacrylamide Tris Borate EDTA (TBE) gel at 4 °C. Retarded samples were transferred to a positively charged nylon membrane then visualized using a stabilized streptavidin detection module (Thermo, 20148 Part No. 89880, 2–5 min exposure).

### DNAI hypersensitivity assay

Cell lines were crosslinked in 0.1% formaldehyde for 10 min with rotation at room temperature and quenched with glycine for 5 min. Cell pellet was resuspended in 1 ml cold 1 × cell lysis buffer (60 mM KCl, 15 mM NaCl, 5 mM MgCl_2_, 10 mM Tris, pH 7.4, 300 mM sucrose, 0.1 mM EGTA, 100 × Halt protease and phosphatase inhibitor cocktail (#PI-78440), 0.1% IGEPAL) and incubated on ice for 10 min. Nuclei were pelleted and resuspended in 50 μl nuclei digestion buffer (60 mM KCl, 15 mM NaCl, 5 mM MgCl_2_, 10 mM Tris, pH 7.4, 300 mM sucrose, 0.1 mM EGTA) per 1 million cell digestion. 5 μl of 100 mM CaCl_2_ was added to each digestion sample and equilibrated at room temperature for 10 min. Samples were incubated at 25 °C for 5 min before DNase I was added and reaction progressed for 10 min at 25 °C. Digestion was terminated with the addition of 350 μl of 0.1 mg ml^−1^ Proteinase K in Nuclear Lysis Buffer (300 mM sodium acetate, 5 mM EDTA, 0.5% SDS). Samples were mixed by inversion and placed in a 55 °C water bath for 5 min and then 65 °C water bath overnight to reverse crosslinks. Additional Proteinase K was added to samples the next day for a final concentration of 0.1 mg ml^−1^ and incubated at 55 °C for 1 h. DNA was isolated with phenol/chloroform/isoamyl (Invitrogen, Cat #15593-031) and chloroform (Amaresco #0757) extractions before ethanol precipitation.

### Computational analyses and statistics

All microarray data were processed and analysed using the R/Bioconductor framework. Specifically, microarray data were processed and normalized using the RMA algorithm and differential expression analysis was performed using the ‘samr' package. Heat map, boxplot and scatter plot visualizations were produced using the ‘pheatmap' package and ‘ggplot2' package, respectively. 20–30,000 CT/TKO Sca1^+^ cells were harvested 2 weeks after Tamoxifen treatment and array analysis were on MOE430 arrays (Affymetrix; GSE63793). In Fig. 2a, we analysed publicly available data generated in CyclinB1-GFP mouse model^15^ and determined the expression of a set of genes in hepatocytes entering acute cell cycle activity upon partial hepatectomy. For Figs 2d and 7d, *P*-value was estimated using a one-sided unpaired *t*-test. For Figs 2c, 3d and 7i,j, *P*-value was estimated using a hypergeometric test. Experiments were repeated until the collection of sufficient data for robust statistical analysis. *P<0.05; **P<0.01; ***P<0.001. Error bars represent standard deviation.

### Data availability

The mass spectrometry data have been deposited in the Dryad database and are accessible at the following URL: http://dx.doi.org/10.5061/dryad.31q7p.

## Additional information

**Accession codes**: Microarray data have been deposited in the Gene Expression Omnibus (GSE63793).

**How to cite this article:** Tarangelo, A. *et al.* Recruitment of Pontin/Reptin by E2f1 amplifies E2f transcriptional response during cancer progression. *Nat. Commun.* 6:10028 doi: 10.1038/ncomms10028 (2015).

## Supplementary Material

Supplementary Information Supplementary Figures 1-7 and Supplementary Table 1

Supplementary Data 1 Different gene programs are activated upon acute or sustained cell cycle activity. Comparison of fold activation for each gene in cycling CyclinB1-GFP+ versus quiescent CyclinB1-GFPhepatocytes (green) and in TKO HCC versus control liver (orange).

Supplementary Data 2 Different gene programs are activated in early and late lesions in TKO HCC. Comparison of fold activation for 17305 expressed genes in Sca1+ TKO cells versus Sca1+ control cells (blue) and in TKO HCC versus control liver (purple).

Supplementary Data 3 The transactivation of a common set of 296 target genes is amplified from early to late lesions in TKO HCC. Comparison of fold activation for the 296 genes in Sca1+ TKO cells versus Sca1+ control cells (blue) and in TKO HCC versus control liver (purple)

Supplementary Data 4 ChIP-Seq analysis of E2f1 binding profile in TKO HCC. ChIP was performed by pulling-down endogenous E2f1 and the pulled-down fraction was analyzed by deep sequencing (30x).

Supplementary Data 5 Interactome of E2f1 and E2f3 in TKO HCC. List of proteins present in the E2f1- and E2f3-pull down fractions in TKO HCC. Proteins are classified by biological functions.

Supplementary Data 6 Genes bound by E2f1 and transactivated in TKO HCC. Intersection of genomic sequences bound by E2f1 (as determined by ChIP-Seq analysis) and genes transactivated in TKO HCC compared to controls (as determined by array analysis).

Supplementary Data 7 Genomic sequences bound by E2F1. Complete results of the ChIP-Seq following precipitation of sheared genomic DNA with an anti-E2F1 antibody.

Supplementary Data 8 Genomic sequences bound by H2a.z. Complete results of the ChIP-Seq following precipitation of sheared genomic DNA with an anti-H2a.z antibody.
