# Supplementary material for: Recruitment of Pontin/Reptin by E2f1 amplifies E2f transcriptional response during cancer progression
Source: Nat Commun. 2015 Dec 7;6:10028. doi: 10.1038/ncomms10028 (PMC4686657; doi:10.1038/ncomms10028)
Supplement: Supplementary Information — Supplementary Figures 1-7 and Supplementary Table 1 [file ncomms10028-s1.pdf]

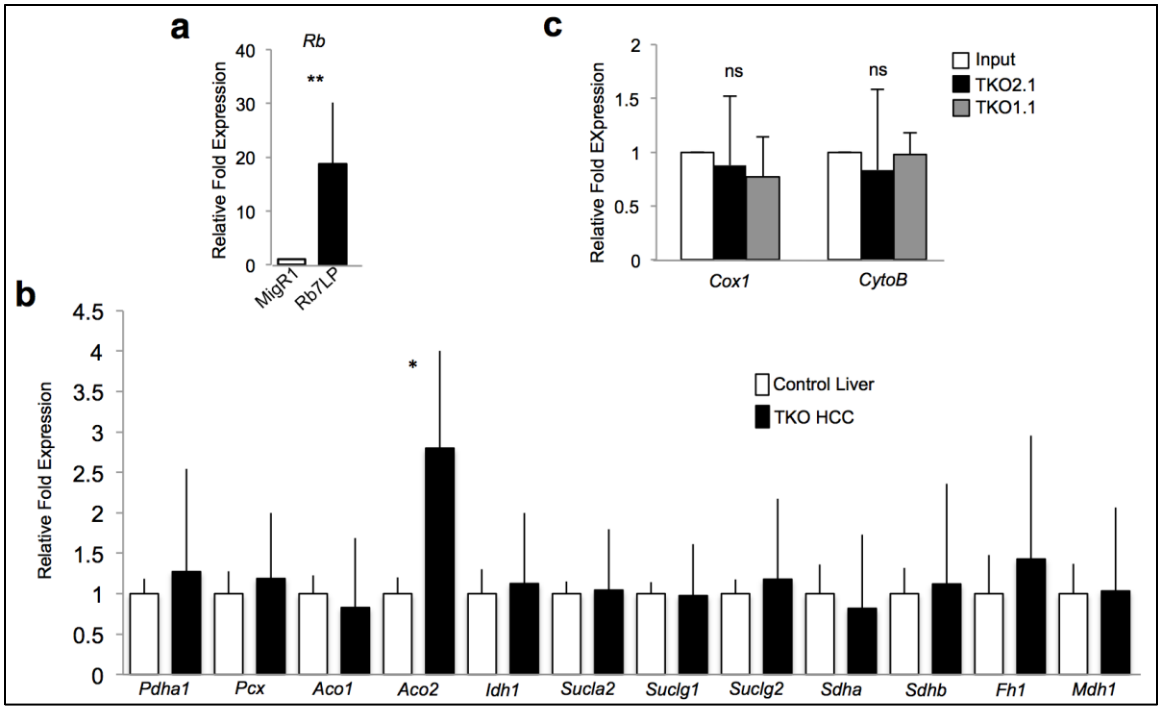

Supplementary Figure 1. E2f factors regulate a set of genes that drive the Warburg effect in TKO HCC. a, Expression of Rb-7LP in TKO HCC cells transduced with either MigR1-IRES-GFP or MigR1-Rb-7LP-IRES-GFP, as detected by RT-qPCR, using primers amplifying a region located in Rb pocket domain (n=3). **b**, Expression of genes involved in the TCA cycle in control liver (n=5) and TKO HCC (n=9), as detected by RT-qPCR. **c,** RT-qPCR analysis to detect the expression of Cox1 and CytoB, two internal markers of mitochondrial biomass, upon expression of Rb-7LP in independent clones of TKO HCC cells (n=3) error bars represent standard deviation. T-test: *: <.05; **: <.01; ***:<.001

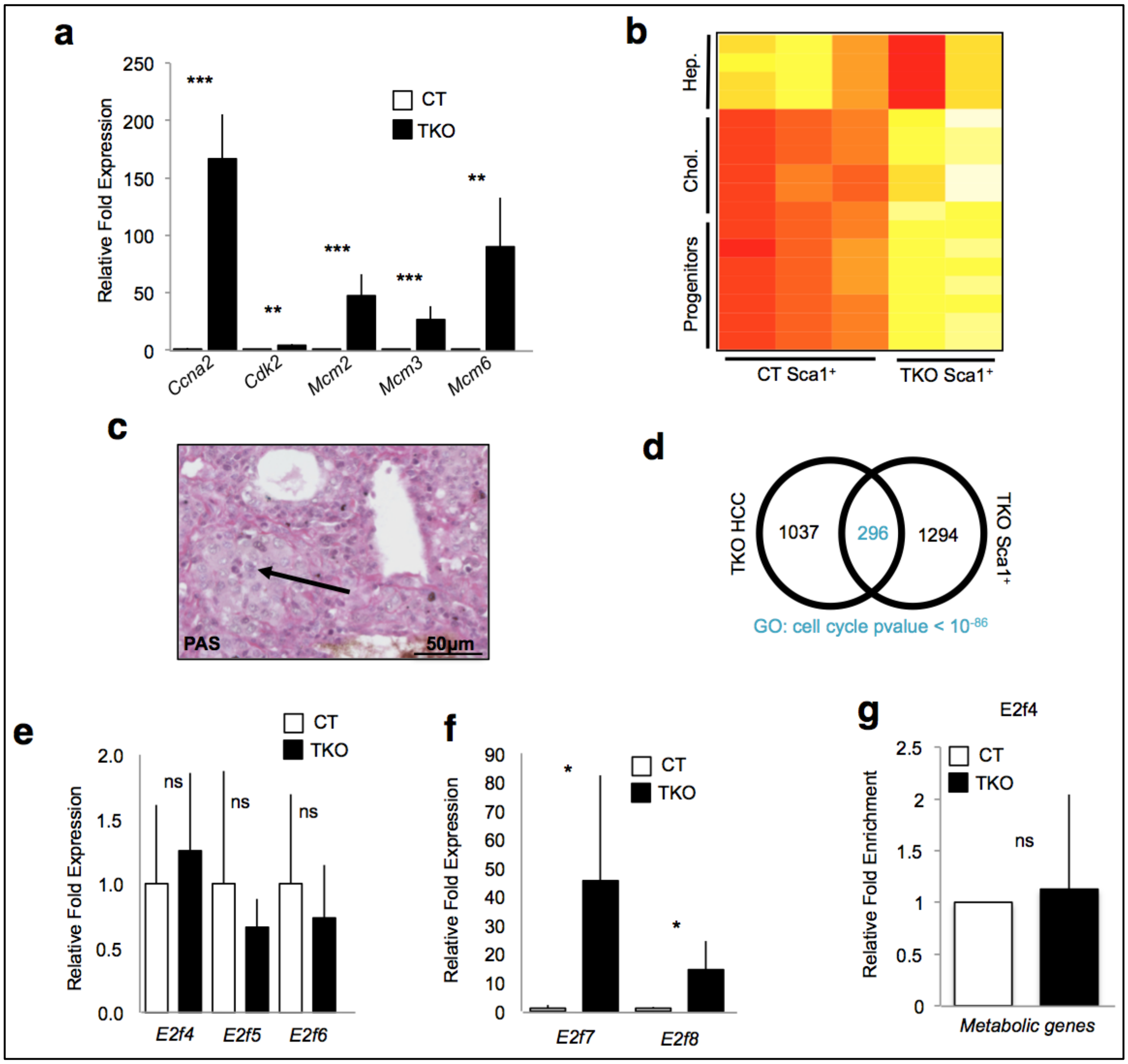

Supplementary Figure 2. Transcriptional amplification of an E2f target geneset from early to late lesions in TKO HCC

a, RT-qPCR analysis in healthy control liver (n=5) and TKO HCC (n=9) for the panel of cell cycle genes used in Fig. 2b (*Ccna2*, *Cdk2*, *Mcm2*, *Mcm3* and *Mcm6*). Note that the transactivation levels are increased compared to transactivation levels observed at 4 days and 12 days after Rb family ablation. **b,** CD31$^-$ CD45$^-$ Ter119$^-$ Sca1$^+$ non-parenchymal liver progenitor cells were isolated from *cTKO* (CT) and *Rosa26-CreER^{T2} TKO* (TKO) mouse 2 weeks after Tamoxifen treatment and processed for RNA array analysis (CT, n=3; TKO, n=2). Expression profile of CT/TKO samples were integrated in a non-

hierarchical clustering analysis (horizontal) and compared with a dataset composed of uncommitted liver progenitors, cholangiocytes and hepatocytes[37]. This analysis determines that CT Sca1$^+$ progenitor cells are uncommitted cells while TKO Sca1$^+$ cells exhibit differentiation features of hepatocytes. **c,** Periodic Acid Schiff (PAS) staining of section from *Rosa26-CreER^{T2} TKO* mice 2 weeks after Tamoxifen. Early lesions still contain glycogen, although certain areas are already devoid of it (black arrow), suggesting a transition between acute and long-term Rb family loss. **d,** Identification of genes transactivated in both TKO HCC (late lesions, left circle) and TKO Sca1$^+$ (early lesions, right circle), compared to their respective controls. Only 296 genes (GO: cell cycle functions, pvalue$<10^{-86}$) are activated in both datasets (pvalue$<10^{-4}$). **e-f**, RT-qPCR analysis of *E2f4-6* (**e**) and *E2f7-8* (**f**) mRNA expression in control livers (n=4) and TKO HCC (n=9). **g**, Binding of E2f4 to the promoter regions of the metabolic target genes in control liver and TKO HCC, as determined by ChIP assay (n=2). An average of the fold enrichment for all metabolic target genes is display. error bars represent standard deviation T-test: *: <.05; **: <.01; ***:<.001

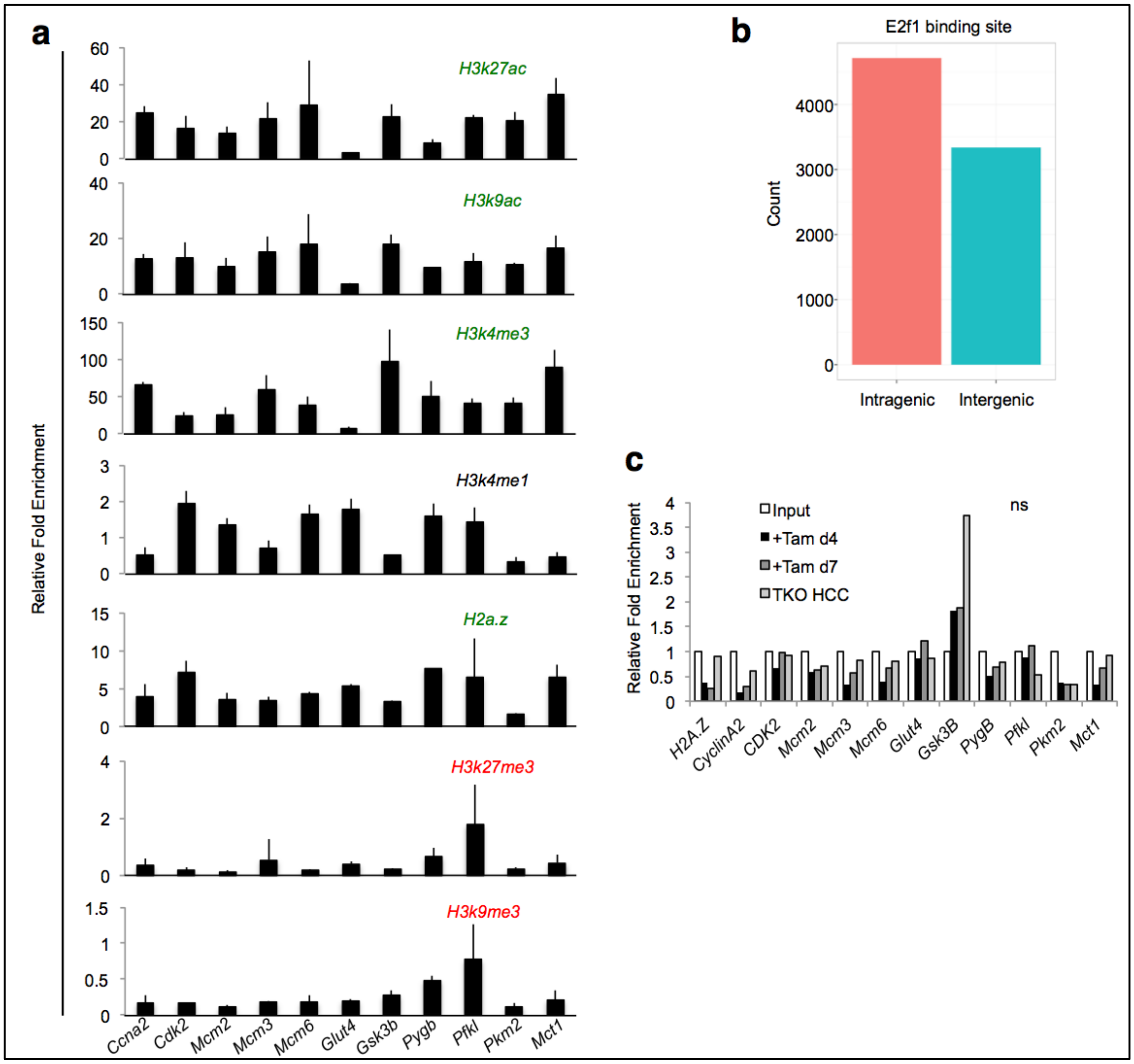

Supplementary Figure 3. Recruitment of a Pontin/Reptin complex by E2f1 in TKO HCC. a, Enrichment for the indicated epigenetic marks at the promoter regions of a panel of representative cell cycle and metabolic target genes in TKO HCC cells. We did not observe any significant difference in the average enrichment between cell cycle and metabolic target genes, suggesting that the variation of transactivation level between both groups (as illustrated in Fig. 2b) is not based on a different epigenetic landscape (n=3). **b**, Intragenic and intergenic location of E2f1 binding in TKO HCC. **c,** Pontin and Reptin can be found either in a hexameric complex or included in larger Tip60 and Srcap complexes, but

components of these complexes were not found in the MS assay (not shown). Tip60 complex displays histone acetyl transferases (HAT) activity performed by the Tip60 protein. Tip60 protein acetylates H2K5ac to facilitate the H2a/H2a.z swapping[21]. To indirectly detect the recruitment of the Tip60 complex by E2f1 in TKO HCC, we performed ChIP assay to detect the enrichment of the H2K5ac epigenetic mark in the promoter region of the panel of E2f target genes at different time points during TKO HCC progression. We found that only *Gsk3b* exhibited modest but non-significant H2K5 acetylation in TKO HCC, which indirectly suggests that the Tip60 complex is not recruited by E2f1 (n=2). T-test: error bars represent standard deviation NS: p-value>0.05.

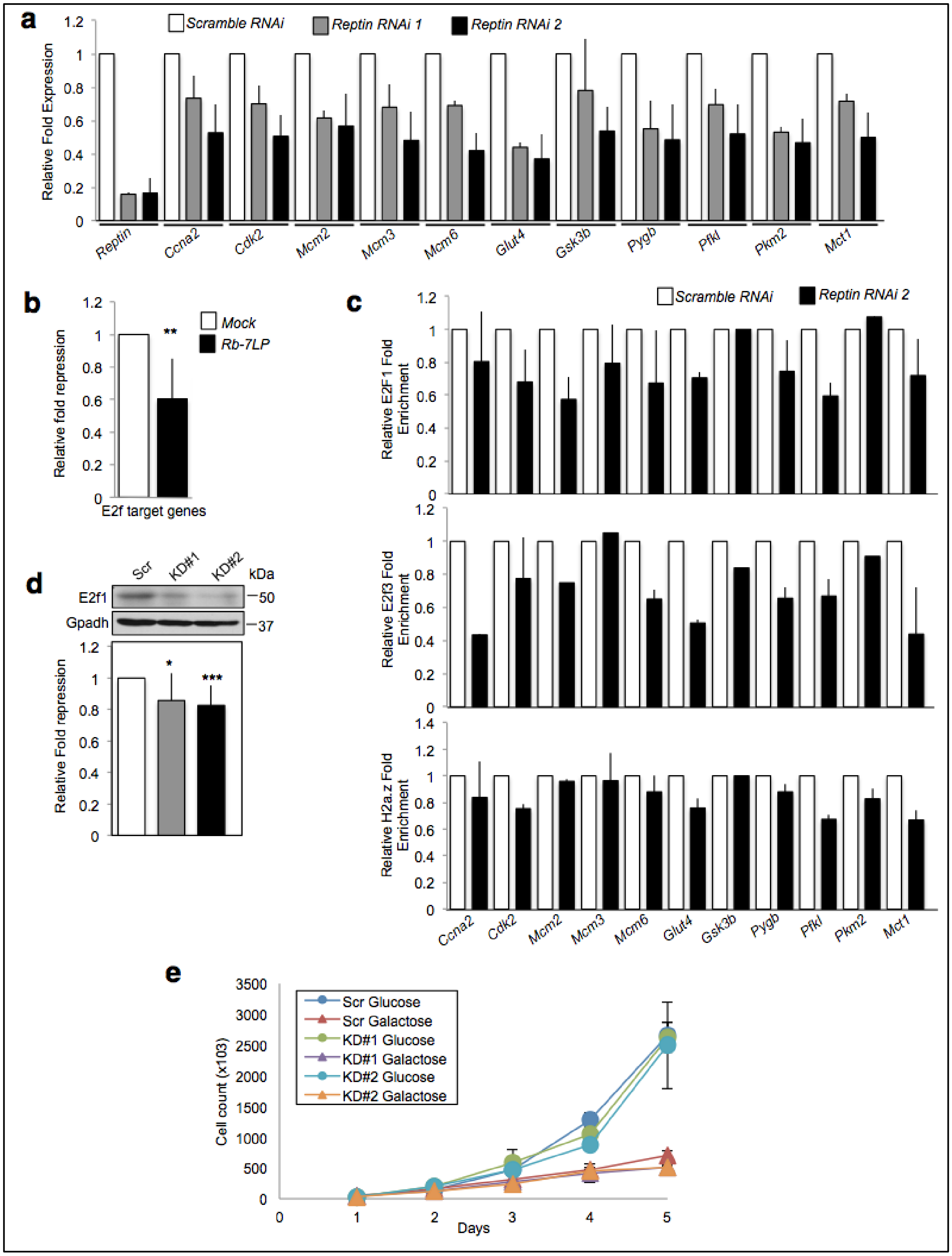

a
Scramble RNAi
Reptin RNAi 1
Reptin RNAi 2
Relative Fold Expression
Reptin
Ccna2
Cdk2
Mcm2
Mcm3
Mcm6
Glut4
Gsk3b
Pygb
Pfkl
Pkm2
Mct1
b
Mock
Rb-7LP
Relative fold repression
E2f target genes
c
Scramble RNAi
Reptin RNAi 2
Relative E2F1 Fold Enrichment
Relative E2f3 Fold Enrichment
Relative H2a.z Fold Enrichment
d
Scr
KD#1
KD#2
kDa
E2f1
Gpadh
50
37
Relative Fold repression
e
Scr Glucose
Scr Galactose
KD#1 Glucose
KD#1 Galactose
KD#2 Glucose
KD#2 Galactose
Cell count (x103)
Days

Supplementary Figure 4. Knock-down of *Reptin* represses the expression of early and late E2f target genes in TKO HCC. a, Expression of *Reptin* and all genes of the panel of E2f target genes (collectively represented as a composite panel in Fig. 5c) upon transfection of TKO HCC cells with *Scramble, Reptin1* and *Reptin2 RNAi* (n=3). **b**, Average repression of *metabolic target genes* upon expression of Rb-7LP in TKO HCC cells, displayed as a composite panel for easier comparison with the average repression of E2f target genes upon *Reptin* silencing displayed in Fig. 5c (n=3). **c**, Enrichment for E2f1 (upper panel), E2f3 (middle panel) and H2a.z (lower panel) at each genes of the panel of E2f target genes (as displayed as a composite figure in Fig. 5d-e) upon silencing of *Reptin* with the *Reptin2 siRNA* in TKO HCC cells (n=3). **d**, Partial knock-down of E2f1 expression in TKO HCC results into limited repression of target genes expression. Knock-down of E2f1 in TKO HCC following the transduction of TKO HCC cells with plko-RFP expressing a scramble and two distinct hairpins to silence *E2f1* (upper panel). This partial knock-down of E2f1 resulted in the limited repression of the expression of the panel of E2f target genes listed in Fig. 2b (n=2). **e**, TKO HCC cells expressing shRNAs either scramble or targeting *E2f1* (KD#1 and #2) displayed similar growth pattern where cultured in the presence of glucose or galactose (n=2). error bars represent standard deviation T-test: *: <.05; **: <.01; ***:<.001

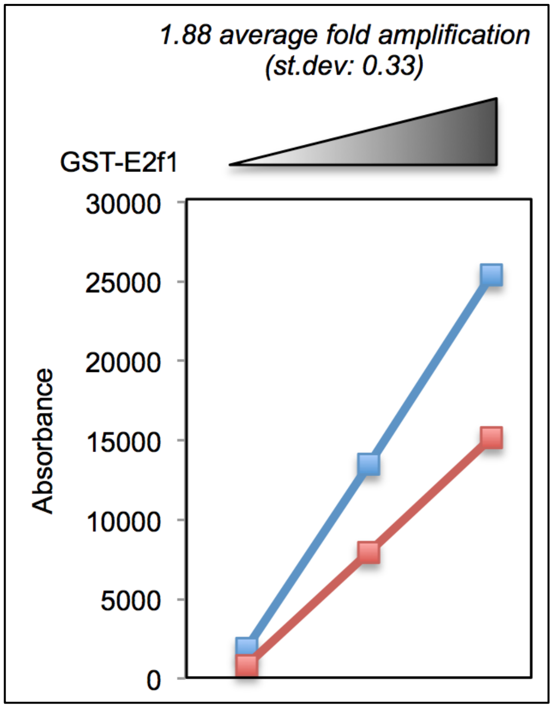

Supplementary Figure 5. Sustained E2f activity transactivates the expression of genes harboring low affinity E2f binding sites in their promoter regions. increased affinity of recombinant E2f1 for the Pfkl T-stretch probe compared to the Pfkl probe. Signals in the gel shift display in Fig. 7h were quantified and display in a graph. The average fold ration at the three doses is 1.88. Quantification of one representative experiment, as displayed in Fig. 7h (n=3).

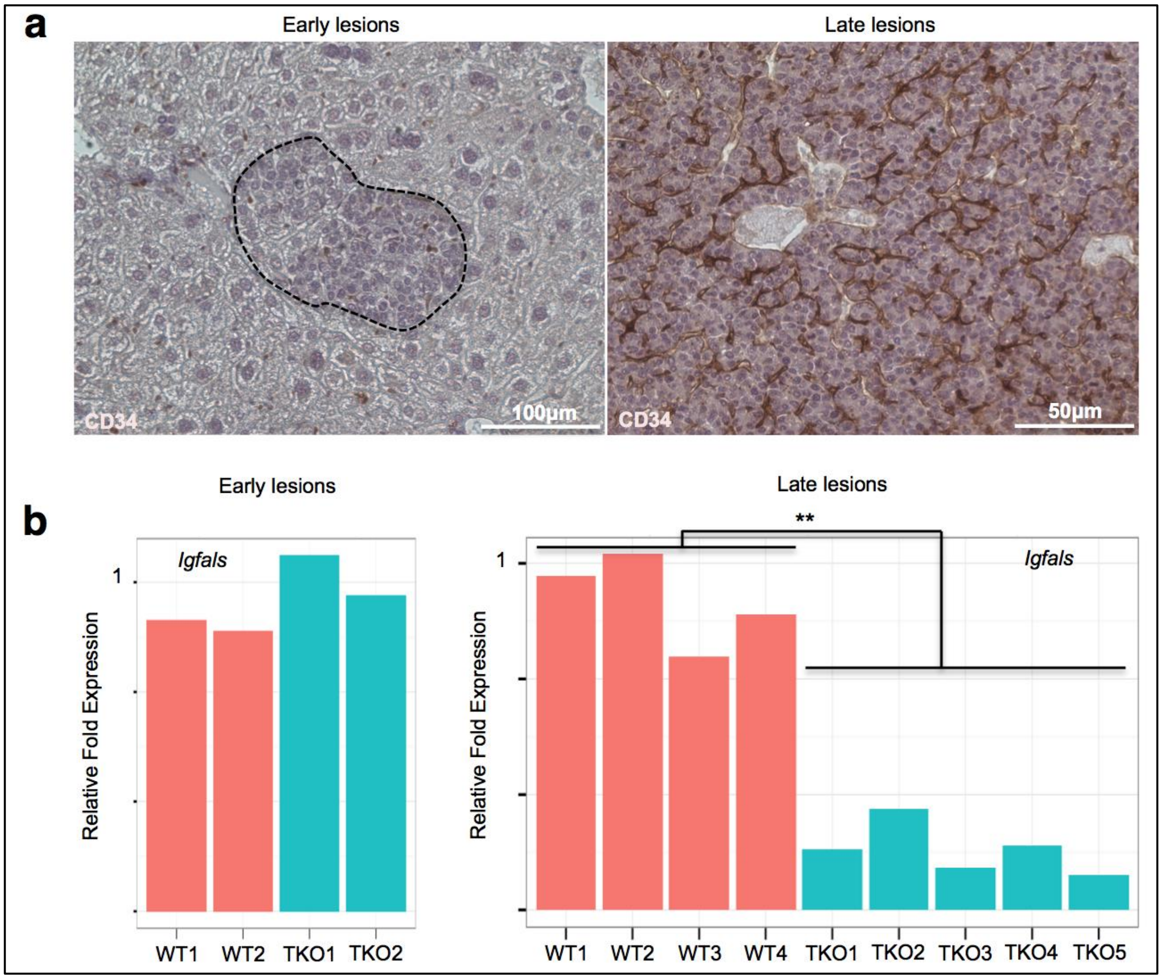

Supplementary Figure 6. CD34 and *Igfals* as relevant markers of TKO HCC progression. **a**, Works by Marquardt et al shows that CD34 expression increases during human HCC progression. Immunohistochemistry staining of early lesions and late lesions with an antibody against Cd34 shows positive staining in late lesions but no staining in early lesions, thereby correlating with the evolution of CD34 expression during human HCC progression. **b**, In addition, the same report demonstrated that the expression of *Igfals* is the single most robust marker of human HCC progression, as the expression of *Igfals* progressively decrease from adenomas to advanced hHCC. Expression of *Igfals* does not vary in early lesions compared to their controls (left) while it decreases in TKO HCC compared to controls (right panels), as determined by array analysis and qPCR (not shown). *: <.05; **: <.01; ***:<.001

Supplementary Figure 7 Uncropped immunoblotting

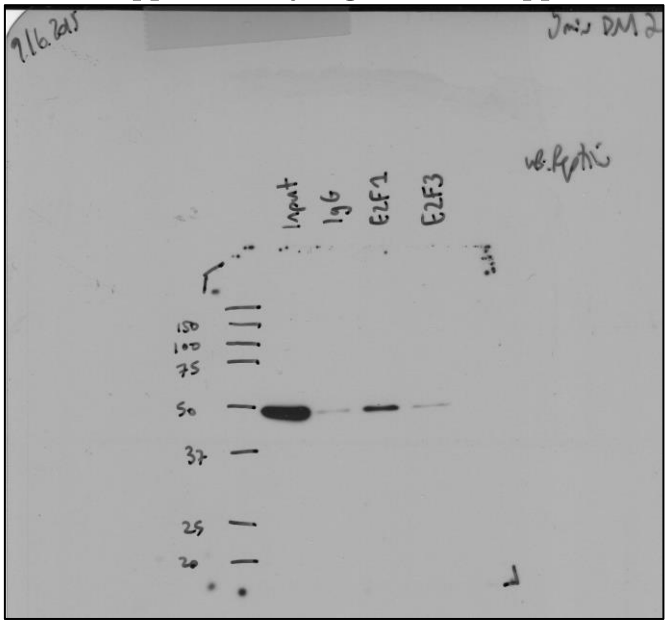

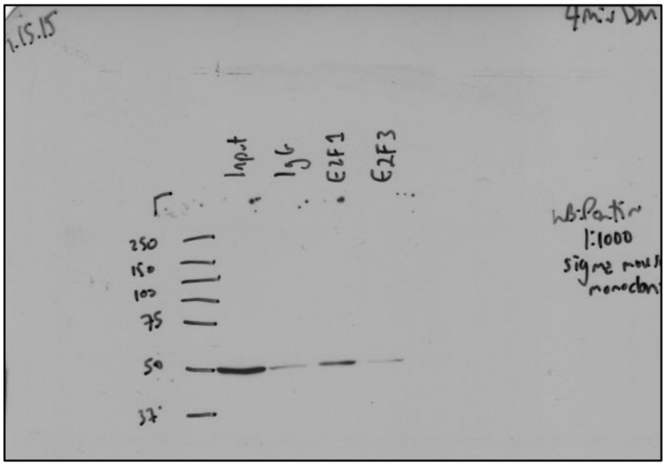

Fig. 4a

Fig. 4c

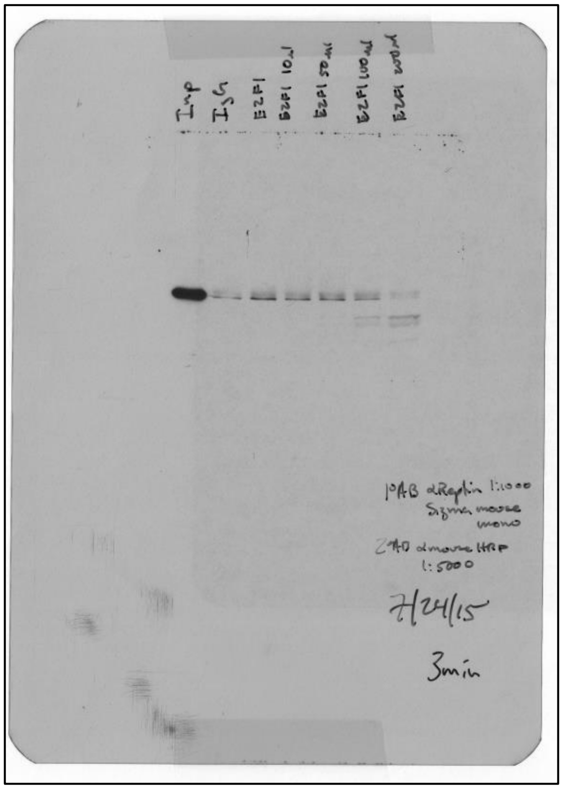

Fig.4g

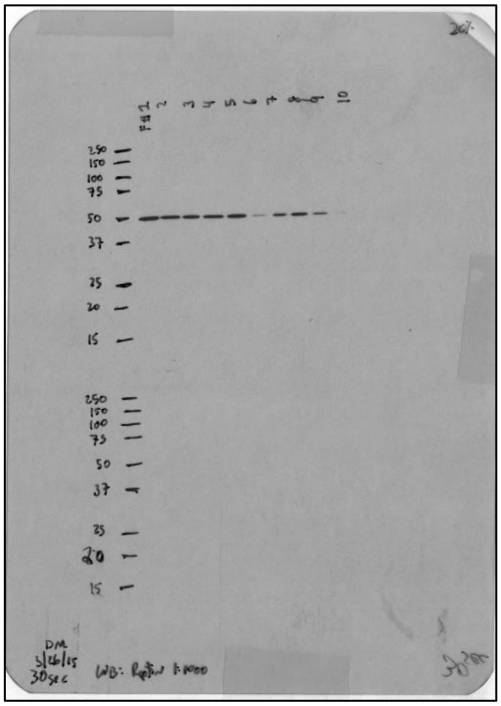

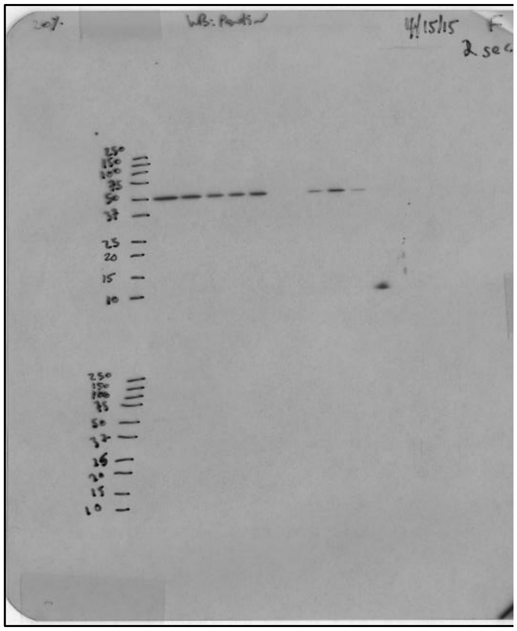

Fig.4h Reptin/Pontin

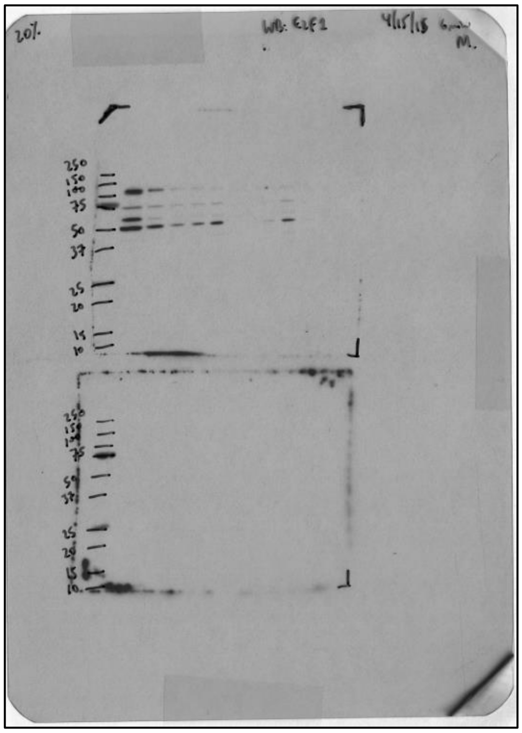

Fig. 4h E2f1

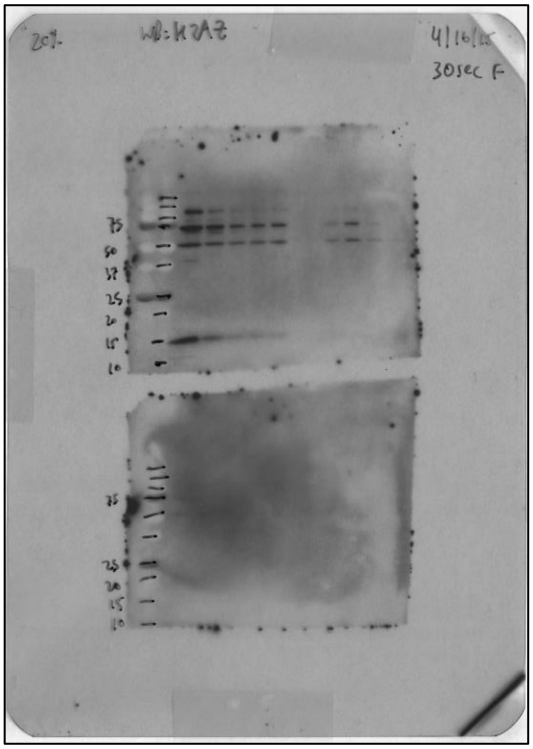

Fig. 4h H2a.z

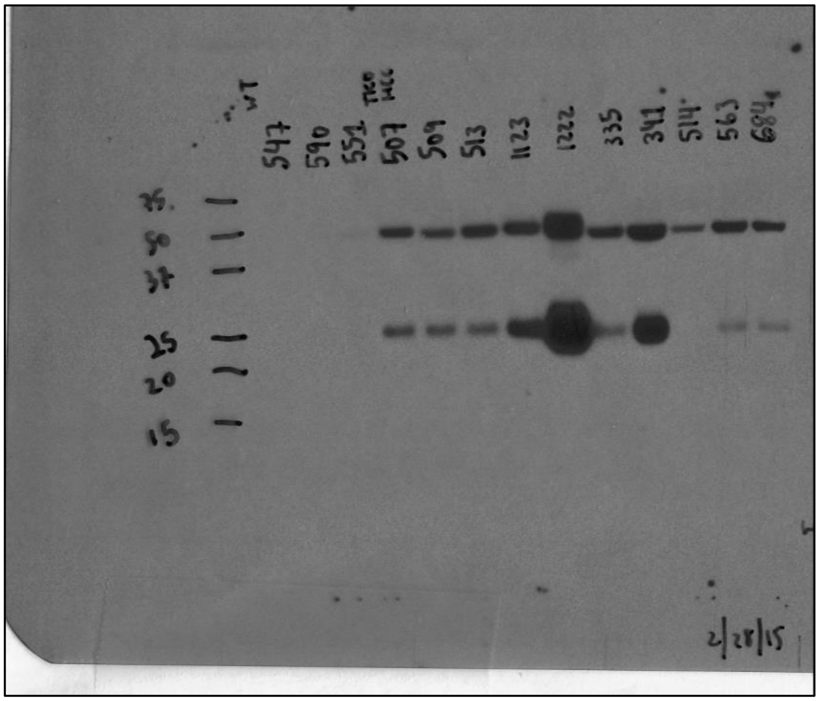

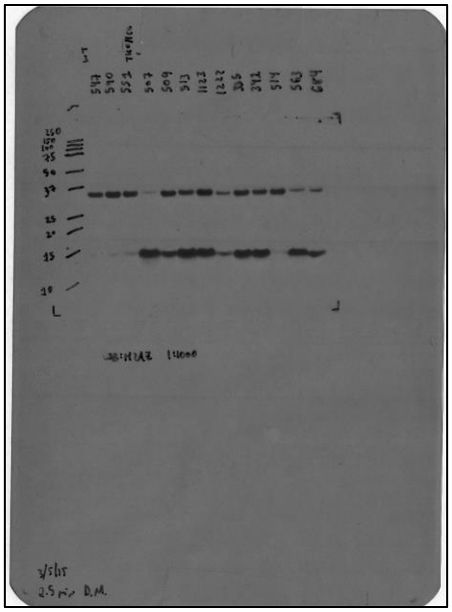

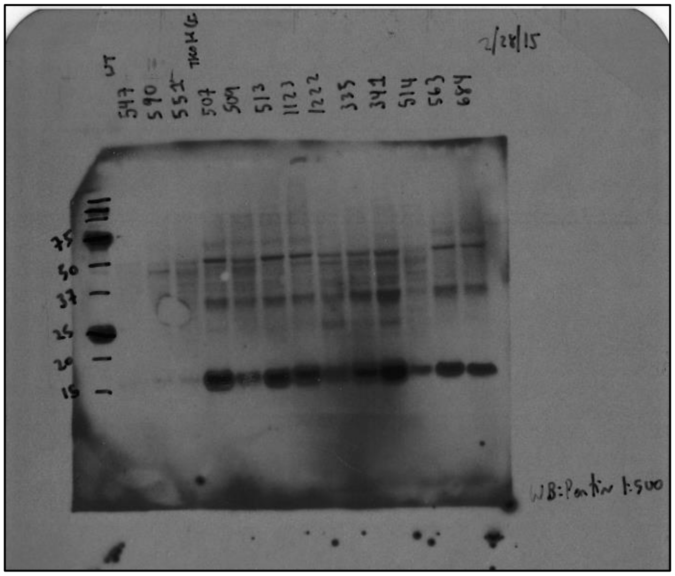

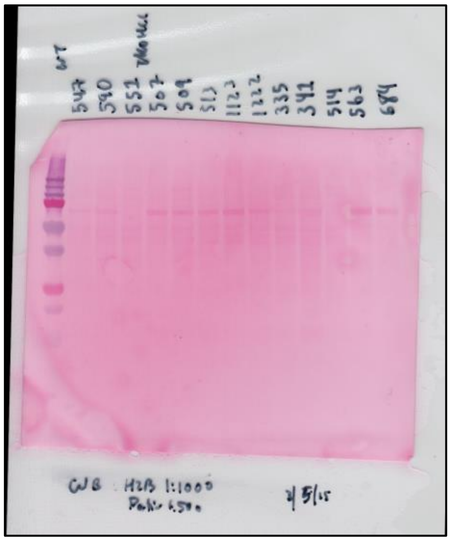

Fig. 6c Reptin/H2a.z/Pontin/Ponceau

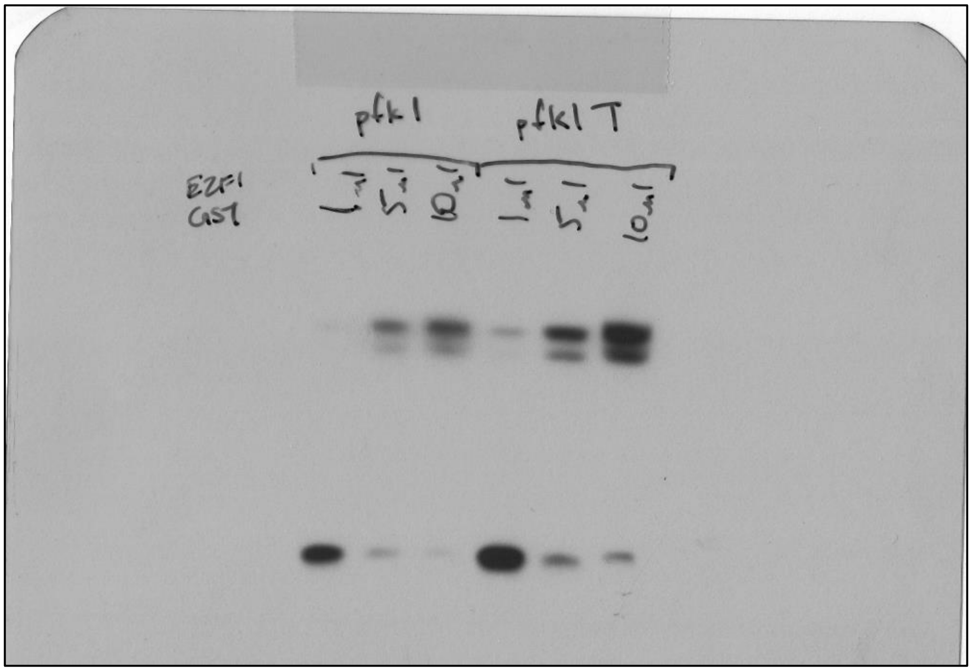

Fig. 7h

Supplementary Table 1. Primers list

Oligo Name		Oligo Sequence	Type
Ccna2	Fwd	CTTGGCTGCACCAACAGTAA	mRNA
	Rev	CAAACTCAGTTCTCCCAAAAACA	
Cdk2	Fwd	GGCTGCATCTTTGCTGAAAT	
	Rev	CCCAGAGTCCGAAAGATCCG	
Mcm2	Fwd	AATCAGGAGGTGAAGCCTGG	
	Rev	CCTGGATGCGGATACGTTGG	
Mcm3	Fwd	AGCCAATCATAACGCGTCTC	
	Rev	CAGCTCCACATCATCCAGCA	
Mcm6	Fwd	ACCTGTACCACAATCTCTGCAC	
	Rev	CACCGCGTTTTACTTCATCA	
Glut4	Fwd	GACGGACACTCCATCTGTTG	
	Rev	GCCACGATGGAGACATAGC	
Gsk3b	Fwd	TTCTACAGGACAAGCGATTTA	
	Rev	CGGACTATGTTACAGTGGTCT	
Pygb	Fwd	GTGGAGCACACCCCTGAC	
	Rev	CGTAGGGCATAGCGAGTAC	
Pfkl	Fwd	CCATGGACGAGGAGAGGTT	
	Rev	TCCAGTTGTTCTCAAAGCTCCT	
Pkm2	Fwd	AAGGGGGACTACCCTCTGG	
	Rev	CCTCGAATAGCTGCAAGTGG	
Mct1	Fwd	GGATATCATCTATAATGTTGGC	
	Rev	GCTGCCGTATTTATTCACCAA	
Pontin	Fwd	CATCGCCCCCATTGTCATCT	
	Rev	TGCCGTGTGGAGAAGTGATG	
Reptin	Fwd	TCATCATGGCCACCAACCGA	
	Rev	TCTCACTGTAGGGTGACGTTG	
H2a.z	Fwd	GGCCGTATTCATCGACACCT	
	Rev	ACTCAAGTACCTCTGCGGTG	

Oligo Name		Oligo Sequence	Type
Ccna2	Fwd	GCCTCTGCCTCCTGAGATG	chIP
	Rev	GAGATGATGTCTCAAAAGTAGAAAAA	
Cdk2	Fwd	TTTAAAGATTCCCAGGTTTTGCTTT	
	Rev	GAACTGGTGGTGTTTCTGGTC	
Mcm2	Fwd	CGAGGGCTCAGCCTTTTGAA	
	Rev	GACGTCACTACGTCCTCGTC	

Mcm3	Fwd	AGCCAATCATAACGCGTCTC	
	Rev	CAGCTCCACATCATCCAGCA	
Mcm6	Fwd	CTCTGGGGGTAAAGGGAGAGA	
	Rev	GCCAAAAGCATACGCCATGAA	
Glut4	Fwd	GGATGGCGGGAAG	
	Rev	GAGACACGCCCCTA	
Gsk3b	Fwd	CAAAAGGAGTGAAAAGCCAAGAGAACG	
	Rev	ATGCAGCATTAAGTTCTCCCGCA	
Pygb	Fwd	CAGCCCACGCCCATCCACAG	
	Rev	ATGCTGCGGTGTCTCTGGCG	
Pfkl	Fwd	CTGGAATCCTAAGGGCCGCCC	
	Rev	CGTGATACGCGATTCCGCCCC	
Pkm2	Fwd	CTACGATGTCCTTCCGCCACGG	
	Rev	TCAGGGTAGGCGGGACAAGCA	
Mct1	Fwd	CCGCGGGTTATAAGGCAGCC	
	Rev	GCACCTACACGACCGACCAGTC	
H2a.z	Fwd	CACGCGCCAATCATCGCTC	
	Rev	TCCTTGAGGCTCCAGCCAAT	
Gapdh	Fwd	TTCACCACCATGGAGAAGGC	
	Rev	CCCTTTTGGCTCCACCCT	

Oligo Name	Oligo Sequence	Type
Early consensus	GNNNNNNTTTTTGGGCGCCNNNNNA	Gel Shift Probes
Pfkl	CAGGCGACAGCGGCGCGGAACGCAG	
Pfkl T-stretch	CAGGCTTTTTCGGCGCGGAACGCAG	

Oligo Name	Oligo Sequence	Type
Reptin Fwd XhoI	CCGCTCGAGCTGGCAACCGTTACAGCC	Cloning
Reptin Rev EcoRI	CCGGAATTCTCAGGAGGTGTCCATGGT	
Pontin Fwd BglII	CCGAGATCTATGAAGATTGAGGAGGTGAA	
Pontin Rev XhoI	CCGCTCGAGTCACTTCATGTACTTATCCT	

Product Name	Cat #	Lot#	ID
Silencer Select Negative Control	4390843	AS00X8E8	
Silencer Select Gapdh siRNA	4390849	AS00XNMG	
Silencer Select Ruvbl2#1 siRNA	4390771	AS00YRR3	s73214
Silencer Select Ruvbl2#2 siRNA	4390771	AS004YRR2	s73215

Oligo Name		Oligo Sequence	Type

Ruvbl2 #1	Sense	GACUGAGGCUGUAACGCAAtt	siRNA
	Antisense	UUGCGUUAGAGCCUCAGUCtt	
Ruvbl2#2	Sense	CGAUGACAUUAAGCGUGUAtt	siRNA
	Antisense	UACACGCUUAAUGUCAUCGac	

Oligo Name		Oligo Sequence
shRNA E2F1 A	Forward	TGCTATGAAACCTCACTAAATTCAAGAGATTTAGTGAGGTTTCATAGCTTTTTTC
	Rev	TCGAGAAAAAAGCTATGAAACCTCACTAAATCTCTTGAATTTAGTGAGGTTTCATAGCA
shRNA E2F1 B	Forward	TGCATTAGAGATCTCTTTGATTCAAGAGATCAAAGAGATCTCTAATGCTTTTTT
	Rev	TCGAGAAAAAAGCATTAGAGATCTCTTTGATCTCTTGAATCAAAGAGATCTCTAATGCA
